# A Metabolic‐Epigenetic Axis of AARS1/IGF2BP3‐K280 Lactylation Sustains Endometrial Cancer Stemness and Recurrence

**DOI:** 10.1002/advs.77757

**Published:** 2026-09-23

**Authors:** Peng Jiang, Yunfeng Zheng, Chenfan Tian, Yuan Tu, Xiaoxia Chang, Jiaxin Yu, Hangkun Yu, Chunxia Gong, Jie Xiong, Philip N. Baker, Yong Fu, Chao Tong

**Affiliations:** ^1^ Department of Obstetrics and Gynecology Chongqing Key Laboratory of Maternal and Fetal Medicine The First Affiliated Hospital of Chongqing Medical University Yuzhong Chongqing China; ^2^ Department of Obstetrics and Gynecology Women and Children's Hospital of Chongqing Medical University Yubei Chongqing China; ^3^ Department of Obstetrics and Gynecology Chongqing Liangjiang New Area People's Hospital Liangjiang New District Yubei Chongqing China; ^4^ Faculty of Medicine and Health Sciences University of East Anglia Norwich Research Park Norwich UK; ^5^ National Clinical Research Center for Child Health and Disorders Ministry of Education Key Laboratory of Child Development and Disorders Children's Hospital of Chongqing Medical University Chongqing China

**Keywords:** AARS1, cancer stemness, endometrial cancer recurrence, IGF2BP3‐K280 lactylation, metabolic‐epigenetic link

## Abstract

Endometrial cancer (EC) recurrence is strongly associated with enhanced tumor stemness. While lactate—a major tumor‐derived metabolite—is known to promote immunosuppression across cancers, its contribution to EC stemness and recurrence remains poorly understood. In this study, we show that elevated protein lactylation in EC correlates with heightened tumor stemness and unfavorable prognosis. Mechanistically, a lactate‐enriched microenvironment induces lactylation of insulin‐like growth factor 2 mRNA‐binding protein 3 (IGF2BP3), thereby stabilizing and upregulating the protein. This modification enhances EC stemness by enabling IGF2BP3 to stabilize HMGA2 mRNA via an m6A‐dependent pathway, activating downstream stemness‐related transcriptional programs. This effect requires lysine 280 (K280) lactylation mediated by alanyl‐tRNA synthetase 1 (AARS1), as either AARS1 depletion or IGF2BP3‐K280 mutation abolishes lactylation and attenuates stemness. Both genetic and pharmacologic inhibition of IGF2BP3 lactylation markedly suppress tumor growth and stem‐like properties in vitro and in vivo. Clinically, IGF2BP3‐K280 lactylation correlates with aggressive clinicopathological features and poor survival, outperforming conventional biomarkers in recurrence prediction. Integration of IGF2BP3‐K280 lactylation into a clinical nomogram substantially improved recurrence‐risk stratification across multi‐center cohorts. Collectively, these findings uncover a metabolic–epigenetic mechanism linking lactate metabolism to m6A‐mediated stemness regulation, establishing IGF2BP3‐K280 lactylation as both a therapeutic vulnerability and a precision biomarker for EC recurrence.

## Graphical abstract

Lactate‐driven endometrial cancer relapse is mediated by enzymatic protein modification. Elevated lactate levels activate AARS1, which catalyzes K280 lactylation of IGF2BP3, therefore stabilizing HMGA2 mRNA and promoting tumor stemness and recurrence. Pharmacological or genetic blockade of AARS1 or IGF2BP3 lactylation suppresses tumor growth. Clinically, IGF2BP3‐K280 lactylation outperforms conventional biomarkers in relapse prediction, supporting its utility in optimized prognostic models and targeted therapies.

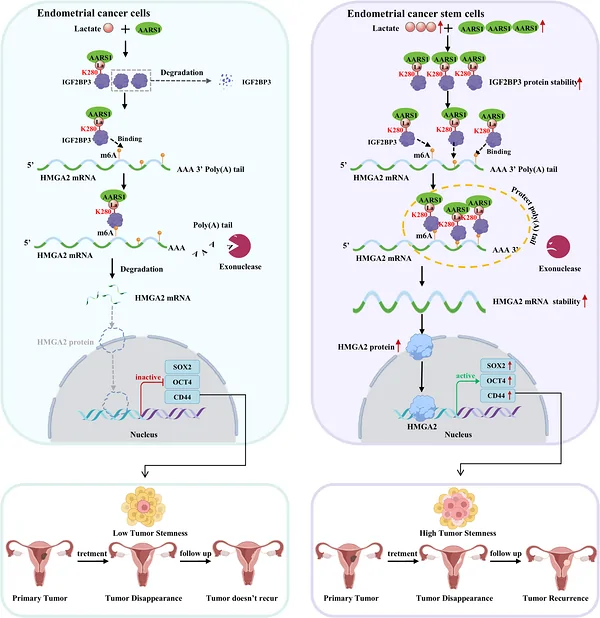

## 1 Introduction

Endometrial cancer (EC) is one of the most prevalent malignancies in the female reproductive system, with its global incidence having surged by 132% over the past three decades [1]. Despite therapeutic advances, disease recurrence remains the primary cause of mortality in EC patients, with suboptimal survival outcomes observed in recurrent cases even after retreatment [2]. Notably, our previous clinical investigations revealed an increasing trend of recurrence rate even among early‐stage low‐risk EC patients [3, 4]. These clinical observations underscore the critical need to investigate novel mechanisms underlying EC recurrence.

Tumor stemness, a fundamental property of cancer stem cells, encompasses self‐renewal capacity, differentiation potential, and tumor‐initiating ability that drives tumorigenesis, heterogeneity, and metastatic progression [5]. In EC, the endometrial cancer stem cells (ECSCs) have been characterized by specific biomarkers including surface antigen (CD133 and CD44), and pluripotency transcription factors (OCT4, SOX2, and NANOG) [6, 7]. Emerging evidence positions tumor stemness as a pivotal contributor to EC pathogenesis and therapeutic resistance, potentially serving as a key determinant of recurrence [8]. Critically, the metabolic reprogramming of ECSCs—particularly lactate accumulation—creates microenvironments permissive to tumor stemness maintenance and recurrence [9], yet the molecular sensors linking metabolism to stemness remain elusive.

Concurrent with metabolic dysregulation, epitranscriptomic modifications—notably N6‐methyladenosine (m6A) methylation—critically modulate cancer progression [10]. m6A dynamics are regulated by writers (e.g., METTL3/14), erasers (FTO, ALKBH5), and readers (e.g., IGF2BP family) [11, 12]. While m6A dysregulation correlates with EC progression [13], its interplay with metabolic reprogramming in regulating EC stemness remains unknown. Specifically, whether metabolic signals post‐translationally modify m6A regulators to enforce stemness programs warrants investigation.

This study reveals a lactate‐AARS1‐IGF2BP3^K280‐lac^/m6A‐HMGA2 axis driving EC recurrence using a multi‐modal approach. Mechanistically, lactate‐induced K280 lactylation stabilizes the m6A reader IGF2BP3 to enhance HMGA2 mRNA stability, promoting stemness. Gemcitabine disrupts this axis to suppress tumorigenicity. Clinically, IGF2BP3^K280‐lac^ serves as a validated recurrence biomarker. We thereby define a druggable metabolic‐epigenetic‐transcriptional cascade for EC recurrence prevention.

## 2 Results

### 2.1 Increased Lactylation and Tumor Stemness in Individuals With Relapsed EC

**FIGURE 1:**
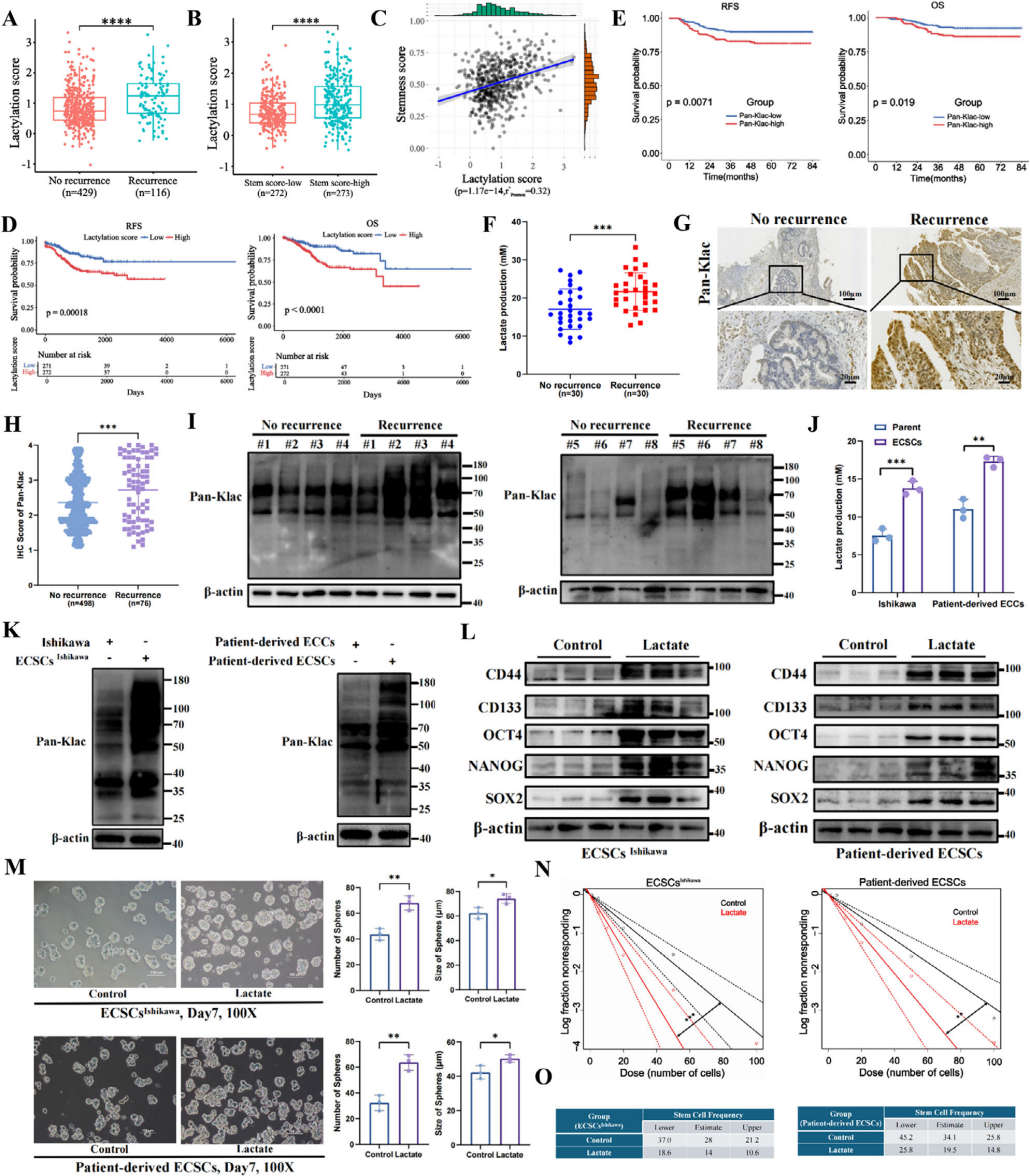
Lactylation modification analysis in ECSCs and recurrent EC. (A,B) Comparison of lactylation scores between non‐recurrent and recurrent EC tissues, and between low and high stemness groups (TCGA‐UCEC; Wilcoxon test); (C) Correlation analysis between lactylation score and stemness score (TCGA‐UCEC; Pearson correlation); (D) Kaplan–Meier analysis of RFS and OS according to lactylation score (TCGA‐UCEC; log‐rank test); (E) Kaplan–Meier analysis of RFS and OS according to lactylation score (institutional cohort, *n* = 574; log‐rank test); (F) Measurement of lactate concentrations in recurrent versus non‐recurrent EC tissues (unpaired *t*‐test); (G) Representative immunohistochemical staining of Pan‐Klac in non‐recurrent and recurrent EC tissues; (H) Quantification of Pan‐Klac IHC scores in non‐recurrent (*n* = 498) and recurrent (*n* = 76) EC tissues (unpaired *t*‐test); (I) Western blot analysis of Pan–Klac in non‐recurrent versus recurrent EC tissues (*n* = 3 replicates); (J) Measurement of lactate concentrations in parental cells (Ishikawa, patient‐derived ECCs) and ECSCs (one‐way ANOVA; *n* = 3 replicates); (K) Western blot analysis of Pan‐Klac in parental cells and ECSCs (*n* = 3 replicates); (L) Western blot analysis of stemness markers in ECSCs treated with or without 20 mm lactate (*n* = 3 replicates); (M) Sphere formation assay of ECSCs treated with or without 20 mm lactate (*n* = 3 replicates); (N‐O) Limiting dilution assay for tumor‐initiating cell frequency in ECSCs treated with or without 20 mm lactate (one‐way ANOVA; *n* = 3 replicates). Data are presented as mean±SD. ^*^, *p* < 0.05; ^**^, *p* < 0.01; ^***^, *p* < 0.001; ^****^, *p* < 0.0001.

We first verified that increased tumor stemness is the core driver of EC recurrence (Figure S1). To address the underlying mechanism, we established ECSC models from multiple EC cell lines and patient‐derived tumors (Figure S2), and selected Ishikawa‐derived ECSCs and patient‐derived ECSCs with the highest intrinsic stemness for subsequent research. We then investigated the role of lactate‐dependent lactylation in this process. TCGA‐UCEC cohort analysis showed that recurrent and high‐stemness EC tumors had significantly higher lactylation levels (*p* < 0.01; Figure 1A, B), and lactylation score was positively correlated with stemness score (r = 0.32, *p* = 1.17 × 10^−^^14^; Figure 1C). Elevated lactylation also predicted unfavorable patient prognosis (*p* < 0.001; Figure 1D, E). These clinical data establish a strong association between lactylation, stemness, and EC recurrence.

We next investigated the source of elevated lactylation. Glycolytic signature analysis of the TCGA‐UCEC cohort revealed elevated glycolytic activity in recurrent and high‐stemness EC patients (Figure S3A,B). Consistently, core glycolytic enzymes (GLUT1, HK2, LDHA) were upregulated in recurrent EC tissues and ECSCs. Moreover, glycolysis score was positively correlated with lactylation score in EC samples (Figure S3C–E), indicating that enhanced glycolysis serves as the primary source of lactate production in high‐stemness recurrent EC. Our clinical cohort further validated increased lactate content and global lactylation levels in recurrent EC tissues (Figure 1F–I). Compared with parental cells, ECSCs displayed higher intracellular lactate accumulation and global lactylation (Figure 1J, K), confirming that ECSC‐specific glycolytic reprogramming induces lactate buildup and lactylation upregulation.

To determine whether lactate directly drives stemness, we performed functional assays. Lactate treatment significantly promoted ECSC stemness, including increased stemness marker expression, sphere‐forming capacity, and tumor‐initiating frequency (Figure 1L–O). In summary, ECSCs exhibit aberrant Warburg effect activation, which enhances glycolysis‐dependent lactate production and protein lactylation, thereby sustaining cancer stemness and driving EC recurrence and progression.

### 2.2 IGF2BP3‐K280 Serves as a Core Lactylation‐Modified Site in ECSCs and Recurrent EC

**FIGURE 2:**
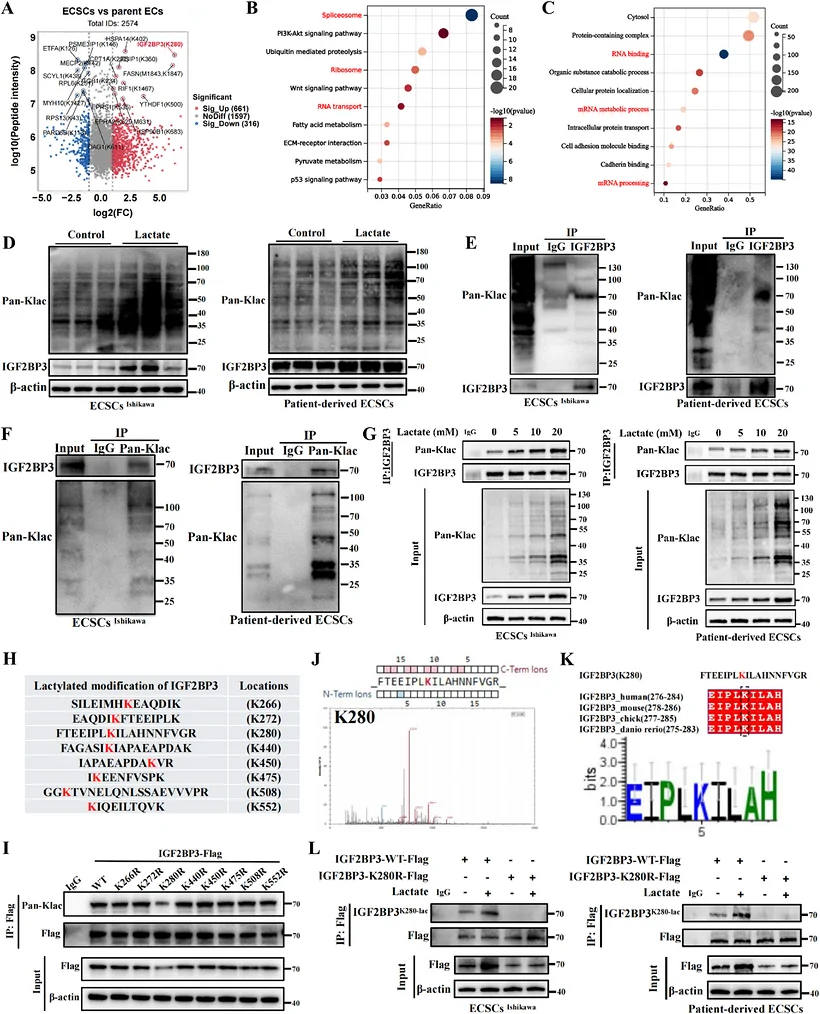
Indentification of IGF2BP3‐K280 as a lactylation site in ECSCs. (A) Scatter plot of lactylation sites identified by lactylome profiling in ECSCs versus parental EC cells (red: upregulated, blue: downregulated); (B,C) KEGG pathway (B) and GO term (C) enrichment analyses of differentially upregulated lactylated proteins in ECSCs; (D) Western blot analysis of IGF2BP3 protein in ECSCs treated with or without 20 mm lactate (*n* = 3 replicates); (E) Co‐immunoprecipitation validation of IGF2BP3 lactylation in ECSCs (*n* = 3 replicates); (F) Reciprocal Co‐IP with pan‐Klac antibody in ECSCs (*n* = 3 replicates); (G) Western blot analysis of IGF2BP3 lactylation and protein stability in ECSCs treated with 0, 5, 10 or 20 mm lactate (*n* = 3 replicates); (H) Lactylome mapping of IGF2BP3 lactylation sites in ECSCs; (I) Co‐IP analysis of IGF2BP3 lactylation in ECSCs expressing IGF2BP3‐WT or K280R mutant (*n* = 3 replicates); (J) MS/MS spectrum of IGF2BP3‐K280 lactylation; (K) Cross‐species conservation analysis of IGF2BP3‐K280; (L) Co‐IP analysis of K280 lactylation in ECSCs expressing FLAG‐tagged IGF2BP3‐WT or K280R with or without 20 mm lactate treatment (*n* = 3 replicates).

Lactylome profiling of ECSCs versus parental cells identified 661 significantly upregulated lactylation sites on 412 proteins, with IGF2BP3‐K280 being a prominently modified residue (Figure 2A). KEGG enrichment analysis of differentially lactylated proteins revealed significant enrichment in stemness‐related pathways (e.g., PI3K‐AKT, Wnt, and ECM‐receptor signaling) as well as RNA regulatory pathways (Spliceosome, Ribosome, and RNA transport) (Figure 2B). Consistent with these findings, GO analysis yielded terms related to RNA binding, mRNA metabolism, and mRNA processing, aligning with IGF2BP3's function as an RNA‐binding protein (Figure 2C). Furthermore, patients with high lactylation scores exhibited elevated m6A levels, and lactylation scores were positively correlated with m6A scores (Figure s4 A,B). Exogenous lactate treatment significantly increased global m6A levels in ECSCs, suggesting that lactylation may regulate stemness through m6A methylation activation (Figure S4C). We therefore focused on IGF2BP3 as a core lactylation target to investigate its role in stemness regulation.

In vitro experiments demonstrated that lactate treatment specifically upregulated IGF2BP3 protein expression in ECSCs without altering mRNA levels (Figure 2D; Figure S4D). Co‐IP assays confirmed robust IGF2BP3 lactylation in ECSCs (Figure 2E, F). Further lactate gradient treatment (0, 5, 10, and 20 mm) demonstrated a dose‐dependent increase in IGF2BP3 lactylation, with a marked increase at 20 mm lactate, accompanied by a significant enhancement of IGF2BP3 protein stability (Figure 2G). These findings indicate that lactate regulates IGF2BP3 expression predominantly through post‐translational lactylation rather than transcriptional regulation. Lactylome mapping identified eight potential lactylation sites on IGF2BP3 (Figure 2H; Figure S5). Site‐directed mutagenesis identified lysine 280 (K280) as a critical site: K280R substitution drastically reduced lactylation and protein levels across multiple species (Figure 2I–K). Using a K280‐lactylation‐specific antibody, we confirmed robust IGF2BP3‐K280 lactylation (IGF2BP3‐K280lac) in ECSCs, which was further enhanced by lactate exposure. In contrast, IGF2BP3‐K280R mutants showed no detectable lactylation or protein recovery with lactate supplementation, underscoring the indispensability of K280 in this regulatory axis (Figure 2L). Altogether, these integrated multi‐omics and functional analyses identify IGF2BP3‐K280lac as a key lactylation event linking lactate metabolism to stemness maintenance in EC recurrence. Furthermore, the observed correlation between lactylation and m6A methylation prompted us to investigate the downstream m6A‑dependent mechanism in subsequent sections.

### 2.3 IGF2BP3‐K280 Lactylation Correlates EC Recurrence and Drives Stemness

**FIGURE 3:**
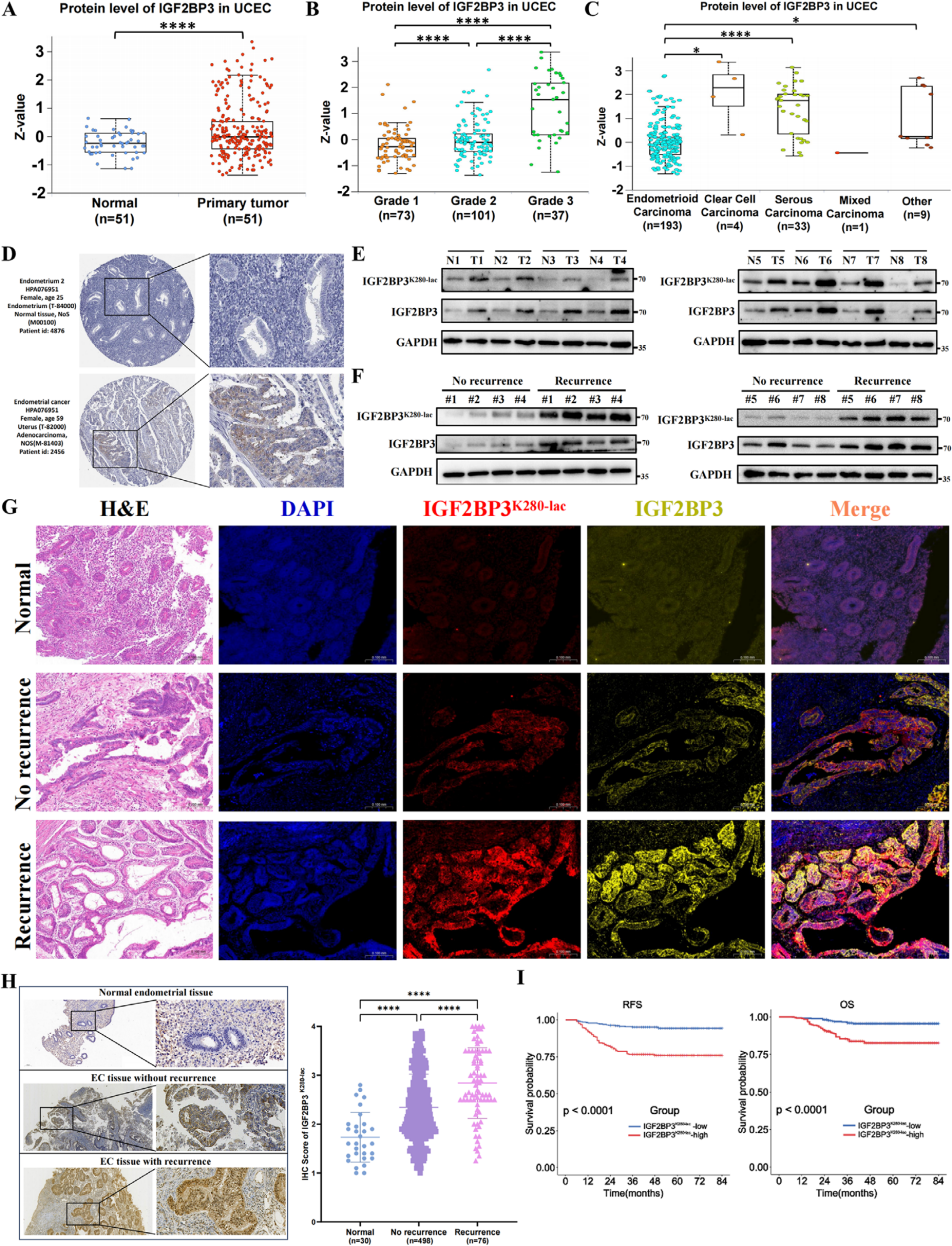
Clinical correlation between IGF2BP3‐K280 lactylation and EC recurrence. (A) Protein expression of IGF2BP3 in tumor versus adjacent tissues (CPTAC database; Wilcoxon test); (B) Protein expression of IGF2BP3 across histological grades (CPTAC database; Kruskal–Wallis test); (C) Protein expression of IGF2BP3 among pathological subtypes (CPTAC database; Kruskal–Wallis test); (D) IHC staining of IGF2BP3 in normal endometrium and endometrial carcinoma (HPA database); (E) Western blot analysis of IGF2BP3‐K280lac and total IGF2BP3 in tumor and adjacent tissues; (F) Western blot analysis of IGF2BP3‐K280lac and total IGF2BP3 in non‐recurrent and recurrent EC tissues; (G) IF staining of IGF2BP3‐K280lac and IGF2BP3 in normal endometrium, non‐recurrent EC, and recurrent EC tissues; (H) IHC analysis of IGF2BP3‐K280lac expression in normal endometrium (*n* = 30), non‐recurrent (*n* = 498), and recurrent EC (*n* = 76) (one‐way ANOVA); (I) Kaplan–Meier analysis of RFS and OS according to IGF2BP3‐K280lac expression (institutional cohort, *n* = 574; log‐rank test). Data are represented as mean±SD. ^*^, *p* < 0.05; ^**^, *p* < 0.01; ^***^, *p* < 0.001; ^****^, *p* < 0.0001.

CPTAC and HPA proteomics data revealed elevated IGF2BP3 expression in EC tissue compared with normal endometrium, with further increases in high‐grade/aggressive tumors (Figure 3A–D). Western blotting in our cohort confirmed this and further demonstrated significantly higher IGF2BP3‐K280lac in EC tissues compared with adjacent normal tissue (Figure 3E). Notably, recurrent EC cases exhibited significantly higher IGF2BP3‐K280lac and total IGF2BP3 levels than non‐recurrent cases (Figure 3F). These results were corroborated by multiplex immunofluorescence, which also revealed significant spatial co‐localization between IGF2BP3 protein and IGF2BP3‐K280lac signals (Figure 3G). Expanding these observations to a larger cohort, immunohistochemical analysis on 574 EC patients from our institution showed that IGF2BP3‐K280lac level progressively increased from normal endometrium to carcinoma tissues and was highest in recurrent cases (Figure 3H). Furthermore, high IGF2BP3‐K280lac level was significantly associated with worse recurrence‐free (RFS) and overall survival (OS) (Figure 3I). Collectively, these analyses establish IGF2BP3‐K280lac as a disease progression‐associated biomarker that correlates with EC aggressiveness and recurrence, and predicts diminished survival.

**FIGURE 4:**
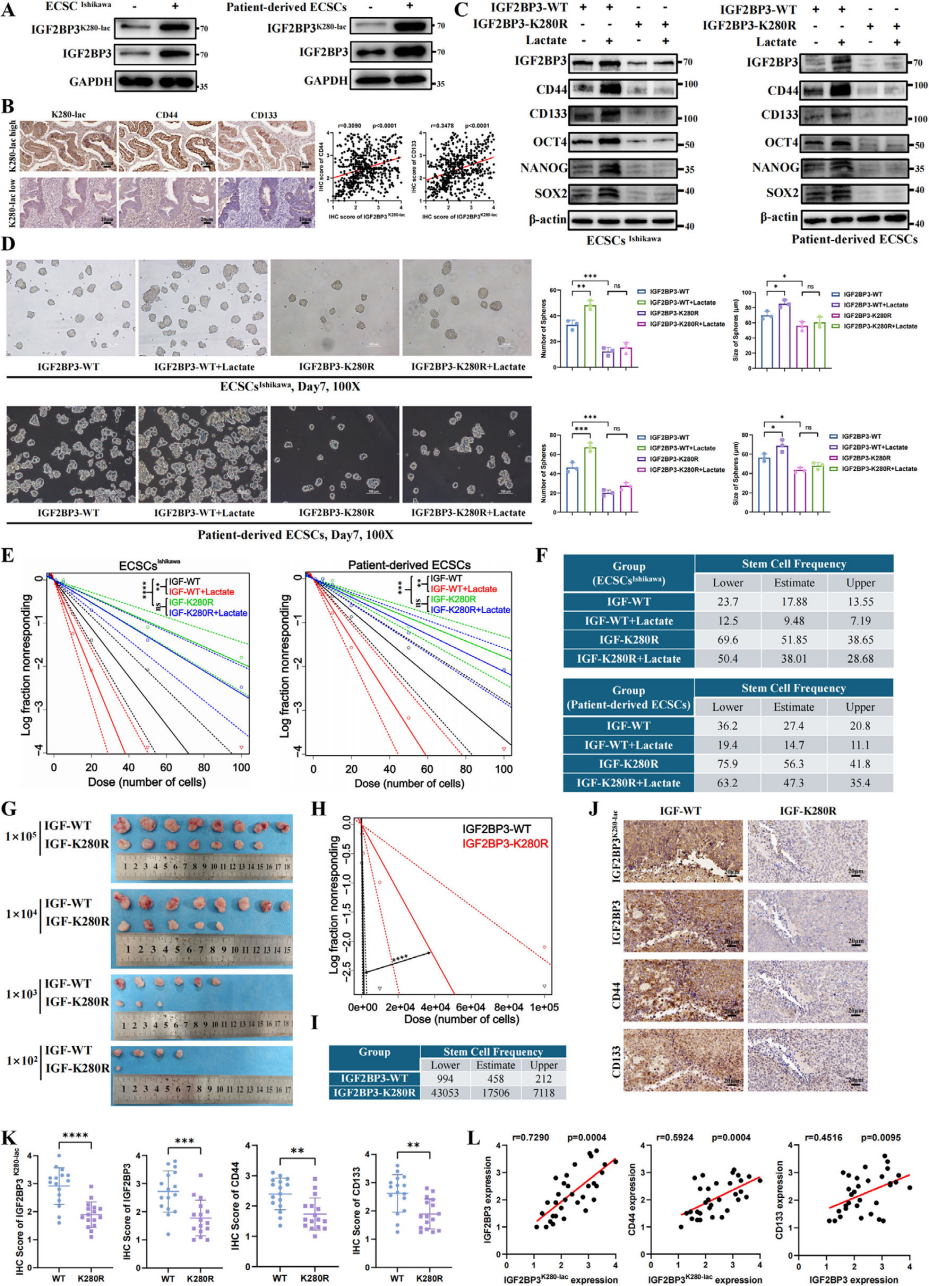
IGF2BP3‐K280 lactylation sustains cancer stemness in EC. (A) Western blot analysis of IGF2BP3‐K280lac and total IGF2BP3 in ECSCs and parental cells (*n* = 3 replicates); (B) IHC analysis and correlation of IGF2BP3‐K280lac with CD44 or CD133 (institutional cohort, *n* = 574; Pearson correlation); (C) Western blot analysis of IGF2BP3 and stemness markers in lactate‐treated ECSCs expressing IGF2BP3‐WT or K280R mutant (*n* = 3 replicates); (D) Sphere formation assay of ECSCs expressing IGF2BP3‐WT or K280R with or without lactate treatment (one‐way ANOVA; *n* = 3 replicates); (E,F) Limiting dilution assay for tumor‐initiating cell frequency in ECSCs expressing IGF2BP3‐WT or K280R with or without lactate treatment (*n* = 3 replicates); (G–I) In vivo limiting dilution assay of ECSCs expressing IGF2BP3‐WT or K280R for xenograft tumor formation (unpaired *t*‐test; K); (J–L) IHC analysis of IGF2BP3‐K280lac, IGF2BP3, CD44, and CD133 in xenograft tumors (Pearson correlation; L). Data are represented as mean±SD. ^*^, *p* < 0.05; ^**^, *p* < 0.01; ^***^, *p* < 0.001; ^****^, *p* < 0.0001.

We nest investigated the functional role of IGF2BP3‐K280lac in regulating cancer stemness. ECSCs exhibited significantly elevated IGF2BP3‐K280lac levels accompanied by increased IGF2BP3 protein expression compared with their parental EC cells (Figure 4A). Correlation analyses of clinical specimens revealed strong positive associations between IGF2BP3‐K280lac and stemness markers CD44 (r = 0.3090, *p* <0.0001 and CD133 (r = 0.3478, *p* <0.0001; Figure 4B). Compared with IGF2BP3‐WT, the IGF2BP3‐K280R mutation significantly reduced the expression of pluripotency markers (CD44, CD133, OCT4, SOX2 and NANOG), impaired spheroid formation capacity, and decreased tumor‐initiating cell frequency (Figure 4C–F). While lactate enhanced stemness features in IGF2BP3‐WT ECSCs, it failed to rescue these properties in K280R mutants (Figure 4C–F). Consistent with in vitro findings, in vivo limiting dilution assays confirmed severely compromised tumor initiation capacity in IGF2BP3‐K280R mutant ECSCs (Figure 4G–I), accompanied by downregulated expression of IGF2BP3, CD44 and CD133 (Figure 4J–L). Taking these findings together, IGF2BP3‐K280lac is positioned as a core regulator of stemness programs that underline clinical EC recurrence.

### 2.4 IGF2BP3‐K280 Lactylation Drives EC Stemness Through HMGA2

**FIGURE 5:**
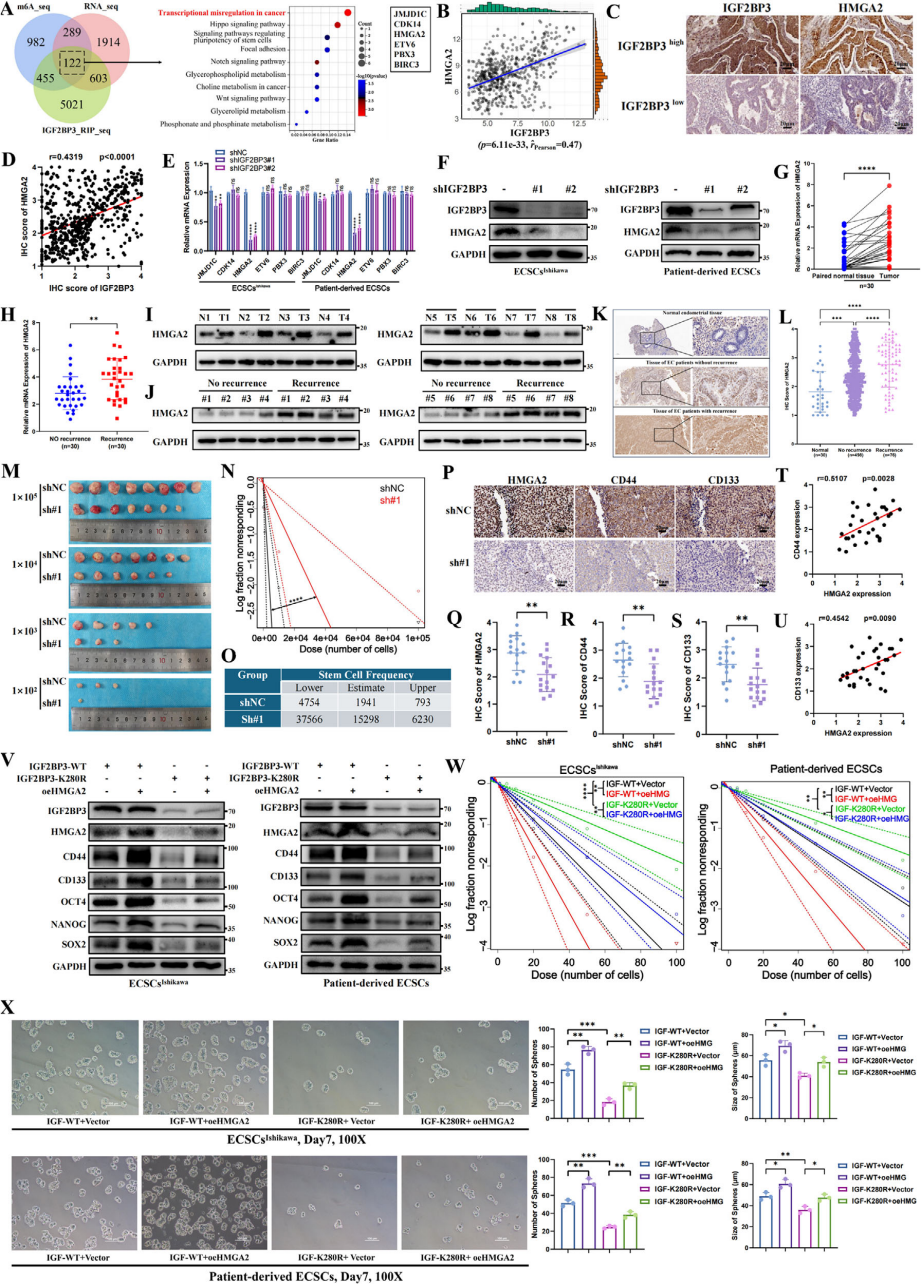
Idenfification of HMGA2 as a downstream target of IGF2BP3‐K280 lactylation in EC. (A) KEGG pathway enrichment analysis of overlapping genes from RNA‐seq, MeRIP‐seq, and RIP‐seq in ECSCs versus Ishikawa cells; (B) Correlation analysis between IGF2BP3 and HMGA2 expression (TCGA‐UCEC; Pearson correlation); (C,D) IHC analysis (C) and correlation (D) of IGF2BP3 and HMGA2 in clinical EC specimens (Pearson correlation; D); (E) RT‐qPCR validation of candidate target genes in ECSCs with IGF2BP3 knockdown (one‐way ANOVA; *n* = 3 replicates); (F) Western blot validation of HMGA2 expression in ECSCs with IGF2BP3 manipulation (*n* = 3 replicates); (G) RT‐qPCR analysis of HMGA2 expression in EC versus adjacent normal tissues (paired *t*‐test); (H) RT‐qPCR analysis of HMGA2 expression in recurrent versus non‐recurrent EC cases (unpaired *t*‐test); (I,J) Western blot analysis of HMGA2 expression in EC versus adjacent normal tissues (I) and in recurrent versus non‐recurrent EC cases (J); (K,L) IHC analysis of HMGA2 expression in normal endometrium (*n* = 30), non‐recurrent (*n* = 498), and recurrent EC (*n* = 76) (one‐way ANOVA; L); (M–O) In vivo limiting dilution assay of ECSCs with or without HMGA2 knockdown for xenograft tumor formation; (P–U) IHC analysis of HMGA2, CD44, and CD133 in xenograft tumors (unpaired *t*‐test; Q‐S; Pearson correlation; T,U); (V) Western blot analysis of stemness biomarkers in ECSCs with HMGA2 overexpression in IGF2BP3‐K280R mutant background (*n* = 3 replicates); (W) In vitro limiting dilution assay of ECSCs with HMGA2 overexpression in IGF2BP3‐K280R mutant background (*n* = 3 replicates); (X) Sphere formation assay of ECSCs with HMGA2 overexpression in IGF2BP3‐K280R mutant background (one‐way ANOVA; *n* = 3 replicates). Data are represented as mean±SD. ^*^, *p* < 0.05; ^**^, *p* < 0.01; ^***^, *p* < 0.001; ^****^, *p* < 0.0001. sh#1 in (M‐S) means shHMGA2#1.

Given that lactylation activates m6A modification (Figure S3A–C) and that IGF2BP3 functions as an m6A reader regulating RNA stability [14, 15], we performed MeRIP‐seq on ECSCs and parental EC cells. MeRIP‐seq analysis of ECSCs revealed extensive m6A remodeling, with 1019 hypermethylated and 1062 hypomethylated peaks predominantly localized to mRNA 3'UTRs (Figure S6). To identify downstream effectors of IGF2BP3 in regulating EC tumor stemness, we integrated RIP‐seq data from Ishikawa cells with ECSC‐specific m6A and transcriptomic profiles, identifying 122 candidate genes enriched in stemness‐associated pathways (Wnt/Notch/Hippo) and transcriptional dysregulation (Figure 5A). Among six genes (JMJD1C, CDK14, HMGA2, ETV6, PBX3, and BIRC3) enriched in the transcriptional dysregulation pathway, HMGA2 emerged as the top candidate, showing the strongest correlation with IGF2BP3 in the TCGA‐UCEC cohort (r = 0.47, *p* = 6.11e‐33; Figure 5B and Figure S7) and clinical validations (r = 0.4319, *p* <0.0001 by IHC/RT‐qPCR/WB; Figure 5C–F and Figure S8).

HMGA2 is a member of the high mobility group (HMG) protein family, which is highly expressed in most human malignant tumors [16]. Studies have demonstrated that HMGA2 promotes EC progression through multiple biological pathways [17, 18]. However, whether HMGA2 regulates EC stemness remains unknown. Integrated analysis of the TCGA‐UCEC cohort and multimodal validation revealed progressive HMGA2 upregulation in EC, correlating with advanced stage, high‐grade histopathology, unfavorable molecular subtypes and recurrent cases. Furthermore, HMGA2 upregulation was associated with higher recurrence rates, as well as lower RFS and OS (Figure 5G–L and Figure S9). HMGA2 overexpression was also significantly correlated with high tumor stemness scores and stemness marker levels (Figure S10A–E). Functional studies demonstrated that HMGA2 knockdown inhibited the expression of tumor stemness markers (CD44, CD133, OCT4, SOX2, NANOG), impaired sphere‐formation capacity, and eliminated tumor‐initiating potential both in vitro and in vivo (Figure S10F–N and Figure 5M–U).

Having established that HMGA2 is direct target of IGF2BP3, we next investigated whether IGF2BP3‐K280 lactylation regulates ECSC stemness via HMGA2. Mechanistic rescue experiments demonstrated that HMGA2 serves as a critical downstream effector of IGF2BP3. Overexpression of HMGA2 significantly reversed the inhibitory effects of the IGF2BP3‐K280 mutant on stemness markers (Figure 5V) and ECSC self‐renewal capacity, achieving 78% rescue efficiency (Figure 5W–X and Figure S11). Collectivelt, these findings establish the IGF2BP3‐K280lac/HMGA2 regulatory axis as a core driver of stemness and recurrence in ECSCs.

### 2.5 IGF2BP3‐K280 Lactylation Stabilizes HMGA2 mRNA by m6A‐Dependent Poly(A) Protection

**FIGURE 6:**
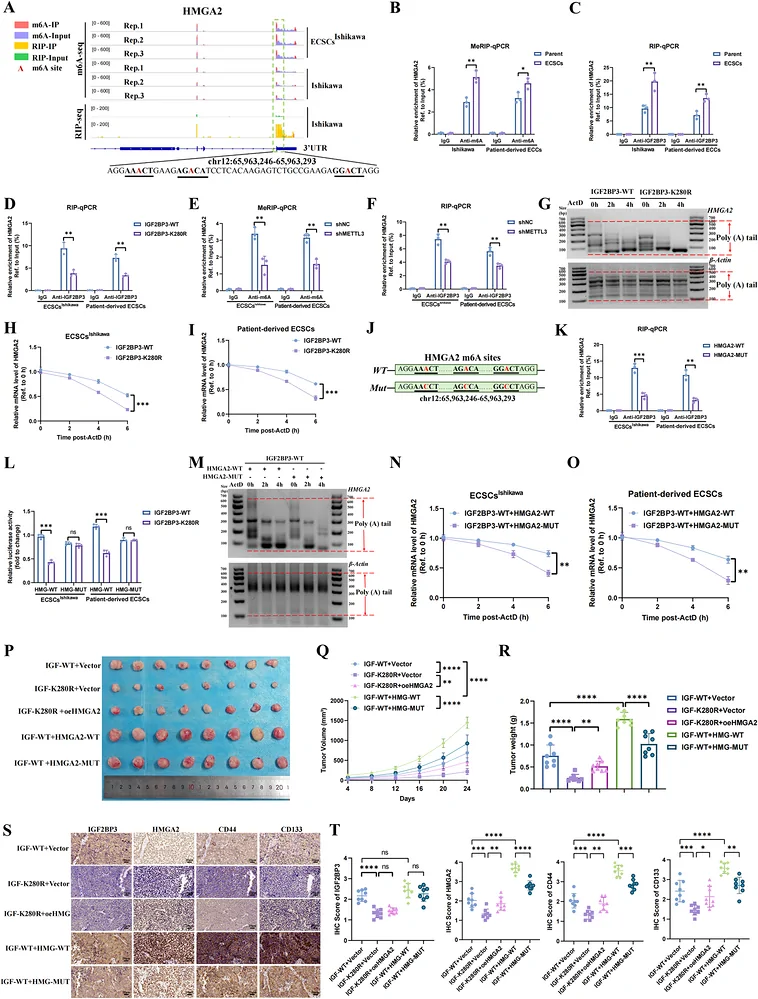
IGF2BP3‐K280 lactylation stabilizes HMGA2 via m6A‐directed poly(A) protection to orchestrate stemness transcription. (A) MeRIP‐seq and RIP‐seq analysis of m6A and IGF2BP3 binding at HMGA2 3'UTR in ECSCs versus Ishikawa cells; (B,C) MeRIP‐qPCR analysis of m6A modification (B) and RIP‐qPCR analysis of IGF2BP3 (C) in ECSCs and parental cells (one‐way ANOVA; *n* = 3 replicates); (D) RIP‐qPCR analysis of IGF2BP3 binding in ECSCs expressing IGF2BP3‐WT or K280R (one‐way ANOVA; *n* = 3 replicates); (E) MeRIP‐qPCR analysis of m6A modification in ECSCs with or without METTL3 knockdown (one‐way ANOVA; *n* = 3 replicates); (F) RIP‐qPCR analysis of IGF2BP3 binding in ECSCs with or without METTL3 knockdown (one‐way ANOVA; *n* = 3 replicates); (G) Poly(A) tail length assay for HMGA2 in ECSCs expressing IGF2BP3‐WT or K280R after actinomycin D treatment; (H,I) RNA stability assay of HMGA2 mRNA in ECSCs expressing IGF2BP3‐WT or K280R after actinomycin D treatment (two‐way ANOVA; *n* = 3 replicates); (J) Schematic of HMGA2 3'UTR m6A site mutation; (K) RIP‐qPCR analysis of IGF2BP3 binding to HMGA2‐WT or mutant in ECSCs (one‐way ANOVA; *n* = 3 replicates); (L) Dual‐luciferase reporter assay of HMGA2‐WT or mutant in ECSCs expressing IGF2BP3‐WT or K280R (one‐way ANOVA; *n* = 3 replicates); (M) Poly(A) tail length assay for HMGA2‐WT or mutant in ECSCs co‐expressing IGF2BP3‐WT (*n* = 3 replicates); (N,O) RNA stability assay of HMGA2‐WT or mutant in ECSCs co‐expressing IGF2BP3‐WT (two‐way ANOVA; *n* = 3 replicates); (P–R) Xenograft tumor growth analysis of ECSCs expressing IGF2BP3‐WT, K280R, with or without HMGA2‐WT or HMGA2‐mutant (one‐way ANOVA; Q‐R); (S,T) IHC analysis and quantification of IGF2BP3, HMGA2, CD44, and CD133 in xenograft tumors (one‐way ANOVA; T). Data are represented as mean±SD. ^*^, *p* < 0.05; ^**^, *p* < 0.01; ^***^, *p* < 0.001; ^****^, *p* < 0.0001.

To determine whether the regulatory effect of IGF2BP3‐K280lac on HMGA2 expression requires m6A modification of HMGA2, we examined the m6A modifications pattern of HMGA2 mRNA using MeRIP‐seq data. MeRIP‐seq identified significantly elevated m6A peaks at the HMGA2 3'UTR, accompanied by increased RNA expression in ECSCs compared with parental EC cells, which colocalized with IGF2BP3 binding peaks at three conserved motifs (AAACT/AGACA/GGACT) (Figure 6A). ECSCs exhibited 2.3‐fold higher m6A enrichment (Figure 6B) and enhanced IGF2BP3‐HMGA2 binding (Figure 6C), both of which were abolished by the IGF2BP3‐K280R mutation (Figure 6D). Critically, METTL3 depletion reduced HMGA2 m6A levels (Figure 6E) and disrupted IGF2BP3 binding (Figure 6F), confirming that this RNA‐protein interaction depends on m6A modification.

While IGF2BP3 is established as an mRNA stabilizer [19], its precise mechanism underlying its stabilizing function remains unclear. Given that the poly(A) tail at mRNA 3'UTRs critically maintains mRNA stability [20, 21] and emerging evidence links m6A depletion near poly(A) tails to accelerated decay [22, 23], we investigated the interplay between m6A modification and poly(A) tails regulation in HMGA2. The IGF2BP3‐K280R mutation markedly accelerated poly(A) tail shortening (Figure 6G) and reduced HMGA2 mRNA half‐life by 46% (Figure 6H,I). Notably, the m6A‐mutant HMGA2 (HMGA2‐MUT; Figure 6J) abolished IGF2BP3 binding (Figure 6K), and dual‐luciferase assays confirmed that IGF2BP3‐K280R significantly suppressed wild‐type (WT) HMGA2 promoter‐driven activity while having no effect on the mutant HMGA2 (Figure 6L). Consistently, HMGA2‐WT rather than HMGA2‐MUT rescue preserved poly(A) length and extended mRNA stability upon IGF2BP3 overexpression (Figure 6M–O), collectively demonstrating that lactylation at K280 is indispensable for m6A‐mediated protection against mRNA decay.

It has been reported that HMGA2 binds to promoter region of target genes and enhances transcription [24]. Thus, we performed ChIP‐seq of HMGA2 in EC cells and found that HMGA2 predominantly binds at the transcription start site (TSS), with strong enrichment for A/T‐rich motifs in promoter regions of stemness‐associated genes (Figure S12A,B). Notably, SOX2, OCT4, and CD44 exhibited pronounced HMGA2 binding peaks at their TSS regions, coinciding with clusters of A/T‐rich regulatory elements (Figure S12C–E). ChIP‐qPCR demonstrated enhanced HMGA2 occupancy at these promoter regions in ECSCs compared with parental EC cells, whereas HMGA2 knockdown abolished the interaction (Figure S12F–K). Functional interrogation through luciferase reporter assay showed that mutating the A/T‐rich motif in SOX2, OCT4, and CD44 promoters completely abrogated HMGA2‐mediated transcriptional activation (Figure S12L–Q). Wild‐type constructs responded to HMGA2 modulation, whereas the mutants showed no response, establishing HMGA2 as a master regulator of stemness through sequence‐specific promoter binding.

To validate the in vivo functional role of the IGF2BP3‐K280lac/HMGA2 axis in EC stemness regulation, xenograft models were established in nude mice. Tumors harboring the IGF2BP3‐K280R mutation exhibited significantly reduced volumes compared with IGF2BP3‐WT controls, a phenotype that was partially rescued by HMGA2 overexpression (Figure 6P–R). Critically, co‐expression of wild‐type HMGA2 with IGF2BP3‐WT increases tumor growth by 210% compared with IGF2BP3‐WT alone, whereas co‐expression with m6A‐mutant HMGA2 results in only 136% enhancement over the same baseline. IHC analysis revealed that ablation of K280 lactylation markedly suppressed CD44 and CD133 expression, which was partially restored by HMGA2 reconstitution. Consistently, wild‐type HMGA2 co‐expression upregulated CD44 and CD133 levels by 2.4‐ and 1.8‐fold respectively, compared with IGF2BP3‐WT alone, but this effect was largely abrogated in the m6A‐mutant HMGA2 group (1.4‐ and 1.2‐fold; Figure 6S,T). Therefore, these results demonstrate that IGF2BP3‐K280lac drives an oncogenic axis that stabilizes m6A‐modified transcripts and, through HMGA2‐mediated transcriptional regulation, enhances cancer stemness.

### 2.6 AARS1 Catalyzes K280 Lactylation of IGF2BP3 to Stabilize IGF2BP3 and Promote EC Stemness

**FIGURE 7:**
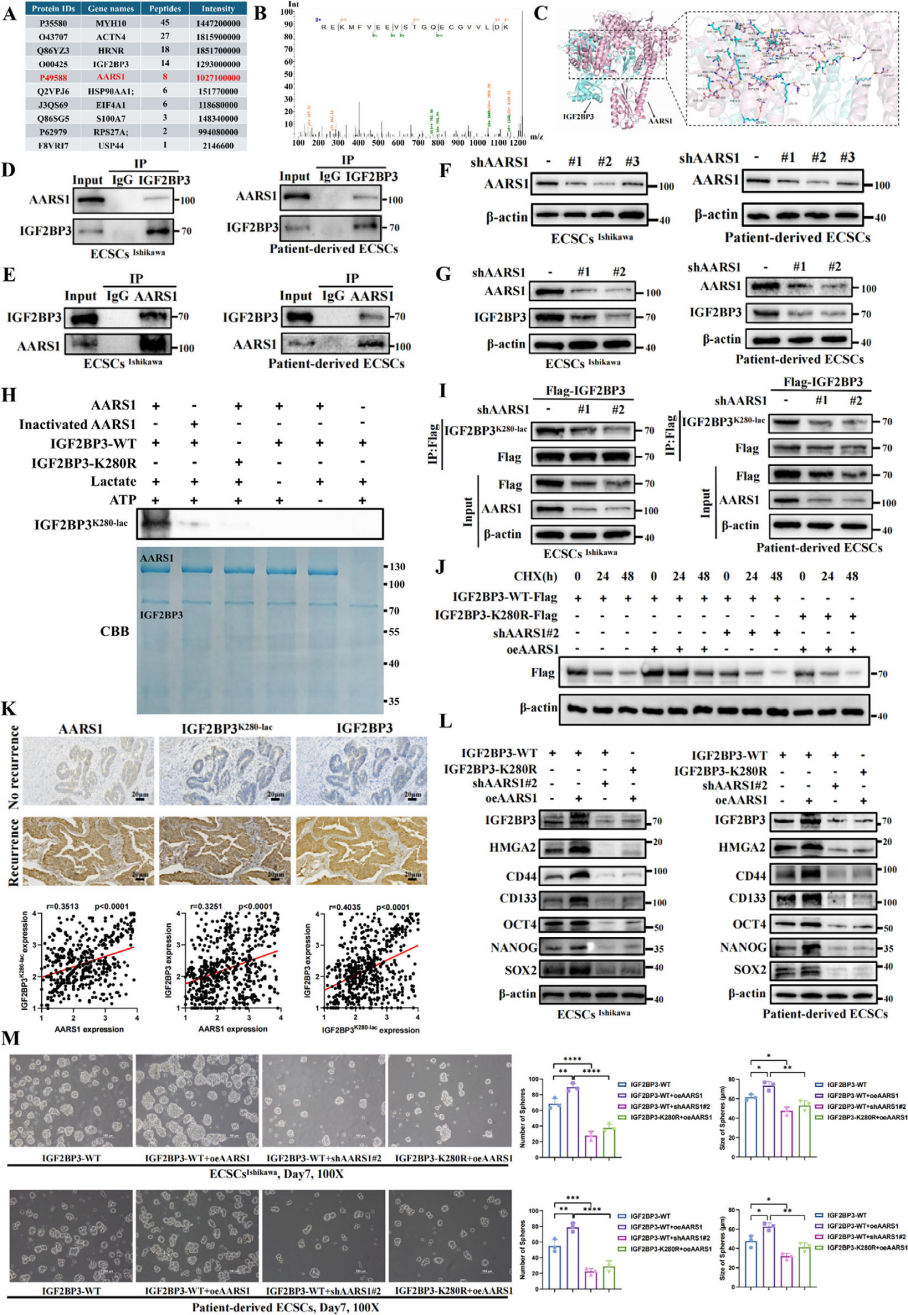
AARS1 catalyzes K280 lactylation of IGF2BP3 to stabilize its protein expression and promote EC stemness. (A) Co‐IP‐MS identification of IGF2BP3‐interacting proteins including AARS1; (B) Representative MS/MS spectra of AARS1 peptides co‐precipitated with IGF2BP3; (C) Protein docking model of AARS1‐IGF2BP3 interaction; (D) Co‐IP analysis of AARS1 with IGF2BP3 in ECSCs (*n* = 3 replicates); (E) Reciprocal Co‐IP of IGF2BP3 with AARS1 in ECSCs (*n* = 3 replicates); (F) Validation of AARS1 knockdown by western blot in ECSCs (*n* = 3 replicates); (G) Western blot analysis of IGF2BP3 expression in ECSCs with AARS1 knockdown (*n* = 3 replicates); (H) In vitro lactylation assay of AARS1‐mediated IGF2BP3‐K280 lactylation (*n* = 3 replicates); (I) Co‐IP analysis of IGF2BP3‐K280 lactylation in ECSCs with AARS1 knockdown (*n* = 3 replicates); (J) Protein stability assay of IGF2BP3‐WT or K280R in ECSCs with AARS1 overexpression or knockdown (*n* = 3 replicates); (K) IHC analysis and correlation of AARS1, IGF2BP3‐K280lac, and IGF2BP3 in EC tissues (Pearson correlation); (L) Western blot analysis of stemness markers in ECSCs with AARS1 manipulation and IGF2BP3‐WT or K280R (*n* = 3 replicates); (M) Sphere formation assay of ECSCs with AARS1 manipulation and IGF2BP3‐WT or K280R (one‐way ANOVA; *n* = 3 replicates). Data are represented as mean±SD. ^*^, *p* < 0.05; ^**^, *p* < 0.01; ^***^, *p* < 0.001; ^****^, *p* < 0.0001.

To identify the enzyme mediating IGF2BP3 lactylation, we performed CoIP‐MS and detected AARS1—a recently identified lactate sensor and lysine lactyltransferase—as a strong IGF2BP3 interactor (Figure 7A, B). Reciprocal CoIP‐MS confirmed the AARS1–IGF2BP3 association (Figure S13), and protein‐docking analyses and CoIP assays further confirmed stable binding that was enhanced by lactate exposure (Figure 7C–E and Figure S14). Although AARS1 is best known as an alanyl‐tRNA synthetase that catalyzes alanine‐tRNA ligation, it also catalyzes lysine lactylation and has been reported to exert oncogenic roles through YAP1 and P53 [25, 26]; however, its involvement in EC was previously unknown. TCGA‐UCEC analysis showed that tumor with high AARS1 expression had higher stemness scores and revealed a positive correlation between AARS1 expression and stemness scores (r = 0.32, *p* = 9.31 × 10^−^^1^ ^5^; Figure S15A, B). Our institutional cohort similarly revealed AARS1 upregulation in ECSCs and recurrent tumors, and this upregulation predicted shorter RFS and OS (Figure S15C–H). These data implicate AARS1 as a driver of EC stemness and recurrence through IGF2BP3 lactylation.

To verify that AARS1 regulates IGF2BP3, we first performed AARS1 knockdown and observed a marked reduction in endogenous IGF2BP3 protein abundance (Figure 7F, G). To further determine whether AARS1 modulates IGF2BP3 through lactylation, we conducted in vitro lactylation enzymatic assays. The results confirmed that AARS1 specifically catalyzes lactylation at the K280 site of wild‐type IGF2BP3. In contrast, mutation of IGF2BP3 at K280, or inactivation or depletion of AARS1, significantly diminished or completely abolished K280 lactylation (Figure 7H). Subsequent cellular experiments further confirmed that AARS1 knockdown simultaneously reduced K280 lactylation levels and IGF2BP3 protein stability (Figure 7I). Protein stability assays further revealed that AARS1 depletion significantly reduced the stability of IGF2BP3‐WT, whereas AARS1 overexpression enhanced IGF2BP3‐WT stabilization. Crucially, this stabilizing effect was abolished in IGF2BP3‐K280R mutant ECSCs, where AARS1 overexpression failed to rescue the compromised stability caused by the K280R mutation (Figure 7J and Figure S16). IHC analysis of our clinical cohort revealed strong positive correlations among AARS1, IGF2BP3^K280‐lac^, and total IGF2BP3 expression (Figure 7K).

Functional assays further demonstrated that AARS1 knockdown significantly diminished expression of stemness markers (CD44, CD133, OCT4, NANOG, and SOX2), reduced spheroid formation capacity, and decreased tumor‐initiating cell frequency in IGF2BP3‐WT ECSCs. Conversely, AARS1 overexpression enhanced these phenotypic effects. Crucially, such enhancement was abrogated in IGF2BP3‐K280R mutant ECSCs (Figure 7L,M; Figure S17). Taken together, these data establish that AARS1 is required for ECSC maintenance by catalyzing K280 lactylation of IGF2BP3, thereby enhancing its stability and stemness‐promoting function.

### 2.7 IGF2BP3‐K280 Lactylation Serves as an Independent Prognostic Biomarker for EC Recurrence

Having established the mechanistic role of AARS1‐catalyzed IGF3BP3‐K280 in driving EC stemness, we next evaluated its translational relevance by assessing its clinical and prognostic significance in EC patients. We assessed IGF2BP3‐K280lac by IHC and correlated its expression with patient characteristics and outcomes. High IGF2BP3‐K280lac expression was significantly associated with older age (mean 54.9 vs 53.1 years, *p* = 0.020), higher FIGO stage (24.9% vs. 18.8%, *p* = 0.088), non‐endometrioid histology (22.1% vs. 11.4%, *p* < 0.001), deep myometrial invasion (34.1% vs. 22.8%, *p* = 0.003), lymphovascular space invasion (32.1% vs. 20.0%, *p* < 0.001), recurrence (22.5% vs. 8.6%, *p* < 0.001), and mortality (16.9% vs. 4.3%, *p* = 0.002; Table S4). Critically, IGF2BP3‐K280lac demonstrated superior predictive performance for recurrence (ROC AUC = 0.697) and death (AUC = 0.675), outperforming those of pan‐lactylation, AARS1, and total IGF2BP3 (Figure S18A,B). Multivariable Cox analysis confirmed IGF2BP3‐K280lac as an independent predictor of recurrence (HR = 3.79, *p* < 0.001) and death (HR = 2.73, *p* = 0.002; Figure S18C,D).

To refine recurrence risk, we constructed a nomogram incorporating IGF2BP3‐K280lac and established clinicopathological prognostic factors—age, FIGO stage, histology, myometrial invasion, LVSI, CA125, and P53 status—using a training cohort (*n* = 574) and validated it in an external cohort (*n* = 231; patient selection criteria: Figure S19; baseline characteristics: Table S5). The model accurately estimated RFS at 1, 3, and 5‐years, with calibration curves showing close agreement between predicted and observed outcomes (Figure S20A,B). Time‐dependent ROC analysis yielded AUCs > 0.80 at all‐time points, peaking above 0.85 for 3‐year RFS (Figure S20C. Adding IGF2BP3‐K280lac significantly improved predictive accuracy over clinicopathologic factors alone (3‐year RFS AUC: 0.882 vs. 0.841 internal; 0.864 vs. 0.805 external; (Figure S20D). Collectively, IGF2BP3‐K280lac is a robust, independent biomarker of aggressive disease and poor survival in EC, and its integration into a validated nomogram markedly enhances recurrence‐risk stratification.

### 2.8 Gemcitabine Inhibits IGF2BP3‐K280 Lactylation to Suppress EC Stemness and Tumor Growth

**FIGURE 8:**
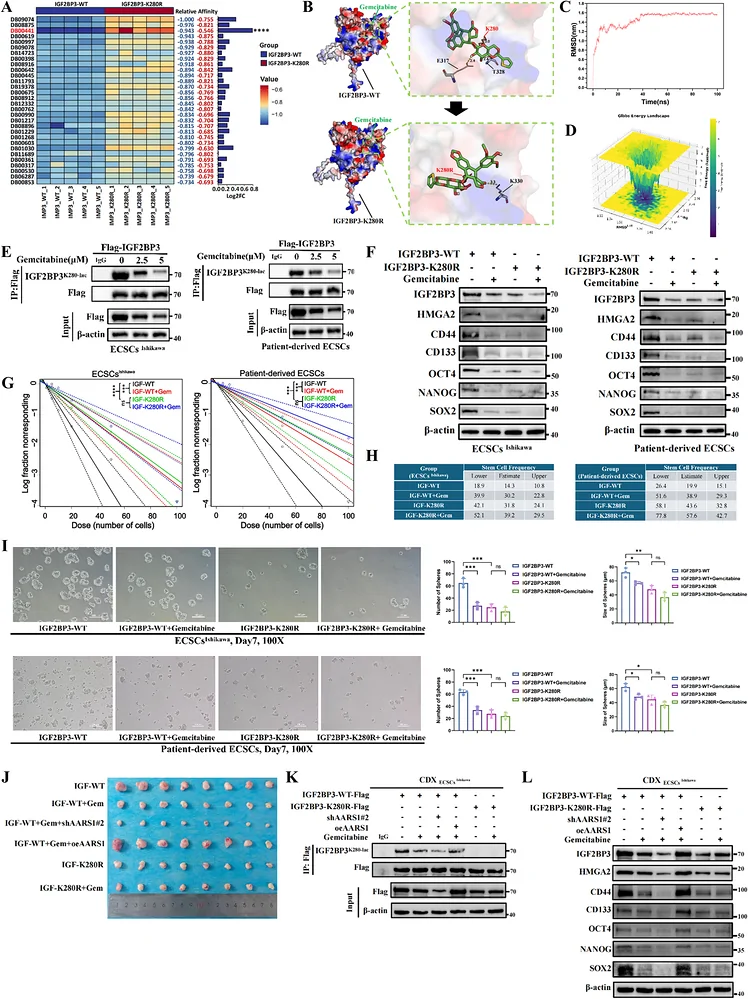
Gemcitabine‐driven suppression of IGF2BP3‐K280 lactylation attenuates stemness traits in ECSCs. (A) Molecular docking analysis of small molecule drugs for binding affinity to IGF2BP3‐WT and K280R mutant; (B) Diagram of gemcitabine docking with IGF2BP3‐WT and K280R; (C,D) Molecular dynamics simulation of IGF2BP3‐gemcitabine complex: (C) RMSD and (D) free energy landscape; (E) Co‐IP analysis of IGF2BP3‐K280 lactylation in ECSCs treated with increasing concentrations of gemcitabine (*n* = 3 replicates); (F) Western blot analysis of stemness markers in ECSCs expressing IGF2BP3‐WT or K280R with or without gemcitabine treatment (*n* = 3 replicates); (G,H) Limiting dilution assay for tumor‐initiating cell frequency in ECSCs expressing IGF2BP3‐WT or K280R with or without gemcitabine (*n* = 3 replicates); (I) Sphere formation assay of ECSCs expressing IGF2BP3‐WT or K280R with or without gemcitabine (one‐way ANOVA; *n* = 3 replicates); (J) Xenograft tumor growth analysis of ECSCs with IGF2BP3‐WT or K280R, combined with AARS1 manipulation and gemcitabine treatment; (K,L) Co‐IP and western blot analysis of IGF2BP3‐K280 lactylation and stemness markers in xenograft tumors (*n* = 3 replicates). Data are represented as mean±SD. ^*^, *p* < 0.05; ^**^, *p* < 0.01; ^***^, *p* < 0.001; ^****^, *p* < 0.0001.

Although the above results establish IGF2BP3‐K280lac as a robust biomarker for EC recurrence, its therapeutic targetability remains to be tested. We therefore performed molecular docking and high‐throughput screening to identify potential inhibitors of IGF2BP3 lactylation. Gemcitabine (DB00441) emerged as a top candidate, showing strong binding to the K280 region of IGF2BP3‐WT (relative affinity = −0.943) but weaker binding to the K280R mutant (−0.546, *p* < 0.0001; Figure 8A). Docking analysis revealed three hydrogen bonds—including one directly with involving K280—that were lost in the K280R mutant (Figure 8B). Molecular‐dynamics simulations confirmed a stable complex (RMSD ∼1.5 nm, RMSF <0.2 nm) with a dominant low‐energy state, progressive structural compaction after 40 ns, and a binding free energy of −16.05 kcal/mol driven by van der Waals and electrostatic forces, supporting gemcitabine as a high‐affinity inhibitor targeting the K280 region (Figure 8C,D and Figure S21).

Based on previously reported studies [27] and our cytotoxicity results (Figure S22), a sublethal dosage of gemcitabine (5 µm) was used in subsequent functional assays. Gemcitabine effectively suppresses IGF2BP3 lactylation while minimizing cytotoxicity‐related non‐specific interference, thereby enabling specific characterization of gemcitabine‐driven epigenetic and phenotypic regulation. Co‐IP assays confirmed that gemcitabine specifically inhibits IGF2BP3‐K280 lactylation in a dose‐dependent manner, achieving ∼70% inhibition at 5 µm and significantly reducing IGF2BP3 protein levels (Figure 8E). In parallel, gemcitabine decreased the expression of core stemness markers (CD44, CD133, OCT4, NANOG, and SOX2) by ∼75% and reduced spheroid formation by ∼59%. Limiting dilution assays further demonstrated a ∼53% reduction in tumor‐initiating cell frequency. These effects were substantially attenuated in IGF2BP3‐K280R mutants (Figure 8F–I).

In xenograft models, gemcitabine monotherapy suppressed IGF2BP3‐WT tumor growth by ∼52%, while combination treatment of gemcitabine with AARS1 knockdown achieved ∼79% inhibition. Conversely, AARS1 overexpression largely abrogated these inhibitory effects (Figure 8J; Figure S23). Compared to WT tumors, K280R mutants exhibited impaired tumor formation, and gemcitabine conferred no additional growth suppression in this context. Mechanistically, gemcitabine inhibited K280 lactylation and stemness marker expression—an effects potentiated by AARS1 depletion but abolished by AARS1 overexpression. In K280R tumors, where lactylation was nearly absent, gemcitabine had no further effect (Figure 8K, L). Together, these computational, cellular, and in vivo results demonstrate that gemcitabine directly targets IGF2BP3 at K280, disrupts its lactylation, and thereby suppresses lactylation‐dependent stemness and tumorigenicity in EC.

## 3 Discussion

Tumor recurrence, predominantly driven by metastasis and therapeutic resistance, relies intrinsically on cancer stemness and represents the leading cause of poor prognosis in EC patients [28]. Accumulating studies have confirmed a tight and direct correlation between tumor metabolic profiles and stemness properties [29, 30]. Here, we found that recurrent EC tissues and high‐stemness EC stem cells (ECSCs) exhibit robust glycolytic activation and upregulated expression of core glycolytic enzymes, which triggers intracellular lactate overload and subsequent protein lactylation, thereby forming a critical glycolysis‐lactate‐lactylation axis that sustains EC stemness. Although glycolysis‐dependent metabolic reprogramming and lactate accumulation are known to facilitate EC progression, the precise mechanism by which this metabolic cascade regulates cancer stemness has remained poorly defined.

Mechanistically, we identify AARS1‐mediated IGF2BP3 K280 lactylation as a vital metabolic sensor that links glycolysis‐derived lactate signals to m6A‐dependent stemness regulation. In recurrent EC and ECSCs, glycolysis‐derived lactate accumulation promotes AARS1‐catalyzed IGF2BP3 K280 lactylation, which stabilizes IGF2BP3 protein and potentiates its biological functions. Lactylation‐stabilized IGF2BP3 further maintains HMGA2 mRNA stability via m6A‐dependent 3'‐UTR binding. While the oncogenic role of IGF2BP3 in tumor metastasis has been previously reported [31], our study is the first to reveal a lactylation‐dependent regulatory mechanism of IGF2BP3 and establishes the IGF2BP3‐K280lac/m6A/HMGA2 axis as the core mechanistic pathway governing EC stemness.

Although HMGA2 has been reported to regulate tumor stemness [32], and to promote EC progression through different biological pathways [18], its role as a downstream target of IGF2BP3 in EC stemness and recurrence has not been established. Here, we further elucidate a novel m6A ‐mediated poly(A) tail maintenance mechanism: IGF2BP3 binding at three consensus motifs (AAACT/AGACA/GGACT) within HMGA2's 3’‐UTR prolongs transcript half‐life by protecting against deadenylation. This expands current understanding of IGF2BP3's RNA‐stabilizing function beyond classical RRACH‐rich element binding [19], revealing epitranscriptomic control of polyadenylation dynamics.

Strikingly, HMGA2 knockdown phenocopied IGF2BP3‐K280R ablation across stemness metrics, with partial phenotypic rescue upon exogenous HMGA2 re‐expression. ChIP‐seq mechanistic dissection demonstrated HMGA2's direct occupancy of A/T‐rich promoter motifs governing core stemness factors (SOX2, OCT4, CD44), extending the known HMGA2‐SOX2 interaction [33] to a comprehensive stemness transcriptional network. This dual regulation—upstream lactylation‐driven protein stabilization coupled with downstream transcriptional amplification—establishes HMGA2 as the master regulator of ECSC homeostasis. Importantly, elevated lactate within ECSC niches creates a self‐reinforcing pathogenic circuit, enhancing AARS1‐mediated IGF2BP3 lactylation to perpetuate feed‐forward stemness programming.

Therapeutically, we identified gemcitabine as a potent axis inhibitor that binds IGF2BP3 near K280, inhibiting lactylation by ∼70% without impairing its inherent RNA‐binding activity. Gemcitabine suppresses ECSC self‐renewal, tumorigenesis, and HMGA2‐driven stemness networks both in vitro (>75% reduction in stemness markers) and in vivo (52% tumor growth suppression)—an effect abrogated by K280 mutation. Beyond providing proof‐of‐concept for targeting the lactylation‐m6A‐stemness cascade, this finding repositions gemcitabine as a metabolic‐epigenetic agent for recurrence prevention. Although gemcitabine is conventionally endorsed in guidelines as a second‐line therapy for endometrial cancer (primarily in platinum/taxane‐resistant or recurrent EC) [2], our study deciphers its novel mechanism through an alternative lens. Crucially, patients with IGF2BP3‐K280lac–positive tumors, particularly recurrent cases, represent a potential subgroup poised to benefit from gemcitabine‐based adjuvant therapy.

Clinically, IGF2BP3‐K280lac expression correlates with aggressive features (advanced stage, LVSI; *p* <0.01) and independently predicts recurrence (HR = 3.791) and mortality (HR = 2.727), with superior prognostic accuracy compared to IGF2BP3 alone (AUC: 0.697 vs. 0.612). Integrated into a multicenter nomogram (*n* = 805), it improved recurrence risk stratification (3‐year AUC = 0.882), positioning IGF2BP3‐K280lac as a functional precision biomarker. Currently, clinicopathological parameters show limited specificity for EC prognosis [1], while molecular classification strategies developed from TCGA data—despite being adopted in guidelines to complement pathological parameters—still require refinement [2, 34], particularly for the large no specific molecular profile (NSMP) subgroup. In this context, IGF2BP3‐K280lac emerges as a promising candidate for further subtyping.

While our study identifies the IGF2BP3‐m6A‐HMGA2 axis and AARS1‐catalyzed K280 lactylation as key regulators of endometrial cancer stemness and recurrence, several limitations to be acknowledged. First, we primarily characterized the AARS1‐lactylation‐IGF2BP3 cascade governing HMGA2 mRNA stability. Despite the involvement of METTL3‐mediated m6A modification in HMGA2 regulation, the interplay between lactylation and the m6A writer machinery has not been fully elucidated and merits further investigation. Second, lactylome profiling was performed exclusively in Ishikawa cells and their corresponding ECSCs, potentially limiting the broad applicability of our omics data. Future studies will include additional endometrial cancer cell lines for lactylome sequencing, aiming to validate and extend our current findings on protein lactylation in cancer stem cells.

In summary, lactate‐driven AARS1/IGF2BP3‐K280lac integrates metabolic dysregulation with m6A‐dependent HMGA2 stabilization to enforce stemness. By therapeutically targeting this lactylation switch and validating IGF2BP3‐K280lac for prognostication, we provide actionable strategies to disrupt the metabolic‐epitranscriptomic drivers of EC recurrence.

## 4 Experimental Section

### 4.1 Patients and Tissue Samples

Thirty paired fresh EC specimens with matched normal adjacent tissues, along with 30 pairs recurrent/non‐recurrent EC tissue pairs were collected from surgically treated EC patients at the First Affiliated Hospital of Chongqing Medical University. Immediately after resection, fresh tissues were washed with saline, frozen in liquid nitrogen and then kept at −80°C for further analysis. Additionally, 805 formalin‐fixed paraffin‐embedded (FFPE) tissue blocks—comprising 574 specimens from our pathology repository (2016–2021 cohort) and 231 specimens from two collaborating tertiary hospitals (Women and Children's Hospital of Chongqing Medical University and Chongqing Liangjiang New Area People's Hospital)—were included for immunohistochemical (IHC) analysis. Comprehensive clinicopathological data encompassing age, body mass index, tumor stage, histotype, grade, myometrial invasion depth, cervical stromal invasion, and LVSI status, were prospectively recorded. Detailed inclusion/exclusion criteria, treatment protocols, and recurrence definition are provided in Supporting Information and our previous publications [35, 36].

### 4.2 Public Database and Associated Analysis Tools

RNA‐sequencing data (raw reads) and clinical data for 545 endometrial carcinoma (EC) cases and 35 normal controls were sourced from TCGA‐UCEC (https://portal.gdc.cancer.gov). Normal endometrial tissue RNA‐seq data (*n* = 142) were obtained from GTEx (https://gtexportal.org). Differential gene expression analysis and visualization were performed using R v4.2.2. Protein expression profiles from patient samples were obtained from the Clinical Proteomic Tumor Analysis Consortium (CPTAC) database. Protein expression profiles underwent two‐step normalization: intra‐sample followed by cross‐sample adjustment of log2 spectral count ratios. Z‐scores were calculated as deviations from the median expression scaled by standard deviations per cancer type. Gene ontology (GO) and Kyoto Encyclopedia of Genes and Genomes (KEGG) pathway analysis were analyzed by Database for Annotation Visualization and Integrated Discovery (DAVID, david.ncifcrf.gov/) online tool and visualized by R software. Single‐gene survival analysis used Kaplan‐Meier Plotter (https://kmplot.com/analysis/).

### 4.3 Analysis of Stemness Score, m6A Score, Lactylation Score and Glycolysis Score

Referring to other similar studies [37], the OCLR algorithm constructed by Malta et al. was used to calculate the mRNAsi (tumor stemness score) of the UCEC dataset. Briefly, this algorithm is based on features from the gene expression profiles of 11,774 genes. Spearman correlation was used to process the RNA expression data, and then the stemness index was mapped to the range of [0,1] through a linear transformation, which involves subtracting the minimum value and dividing by the maximum value. All the above analysis methods were implemented using R software.

To quantify m6A RNA modification status in the UCEC cohort, we developed an m6A score based on 20 key m6A regulatory enzymes, adapting established dimensionality reduction approaches [38]. Briefly, principal component analysis (PCA) was performed on this enzyme set. Scores for the first two principal components (PC1 and PC2) were extracted as feature values. The final m6A score for each patient was defined using a method similar to GGI [39]: m6A score = ∑(PC1_i_+PC2_i_), where the i denotes each m6A enzyme. Similarly, a lactylation score was derived using PCA on 17 major lactate‐modifying enzymes, calculated identically (Lactylation Score = ∑(PC1_j_ + PC2_j_)), with j indexing lactylation enzymes. All the above analyses were implemented using GraphPad Prism 8.0.2 (GraphPad Software, La Jolla, CA).

Consistent with previous studies [40], a glycolysis score model was constructed for UCEC patients. Three glycolysis‐related gene sets were downloaded from the MSigDB database of the GSEA official website. Univariate Cox regression and LASSO regression analyses were sequentially performed to explore the correlation between glycolysis‐related gene expression and overall survival. Genes with *p* < 0.05 in univariate Cox analysis were identified as candidate prognostic genes and further screened via LASSO penalized regression. Ultimately, a prognostic risk score model for EC was established based on the expression levels of screened mRNAs and their corresponding multivariate regression coefficients. The calculation formula was defined as: Risk Score = ∑ [Coefficient(mRNA_k_) × Expression(mRNA_k_)].

### 4.4 Cell Cultures and Treatment

Ishikawa, HEC1‐A, and RL952 cell lines (obtained from the Chinese Academy of Sciences, Shanghai, China) were maintained in DMEM/F12 medium (Gibco, CA, USA) supplemented with 10% fetal bovine serum (FBS, S711‐001S, Lonsera) and 1% penicillin/streptomycin (Gibco, CA, USA). Following methods described in similar studies [8, 41], ECSCs were isolated from these cell lines and cultured in 6‐well ultra‐low attachment plates (Corning, NY, USA). The culture medium consisted of serum‐free DMEM/F12 supplemented with 10 ng/mL human fibroblast growth factor (hFGF; Peprotech, Rocky Hill, USA), 20 ng/mL human epidermal growth factor (hEGF; Peprotech, Rocky Hill, USA), and 2% B27 supplement (Gibco, CA, USA). Growth factors were replenished every 48 h. Sphere formation was monitored using an optical microscope (IX70, Olympus, Japan) on days 3, 5, and 7 post‐isolation. Spheres established by day 7 were harvested for validation of stemness markers via RT‐qPCR, Western blotting, and flow cytometry analysis.

To enhance clinical relevance, patient‐derived endometrial cancer stem cells (patient‐derived ECSCs) were generated in accordance with established protocols. Briefly, fresh EC tissues from patients with late‐stage, metastatic, or high‐grade histological subtypes were dissociated into single cells using type IV collagenase. These cells were then induced into patient‐derived ECSCs and characterized using the same induction and validation methods described above for the cell line‐derived ECSCs (referring to the isolation, culture, and sphere validation procedures mentioned previously). All cell cultures were maintained at 37°C in a humidified atmosphere with 5% CO_2_. All cell lines were verified via short tandem repeat profiling. The Cell Culture Contamination Detection Kit (Thermo, USA) was used to confirm that the cells were free of Mycoplasma contamination. In all experiments, ECSCs were used within 20 passages. Additionally, cells were treated with 20 mm lactate [42, 43] (L6402, Sigma, USA) or 5 µm gemcitabine [27] (HY‐17026, MCE, NJ, USA).

### 4.5 Immunohistochemical (IHC) Staining

Paraffin‐embedded tissues were sectioned at 3 µm thickness, deparaffinized in fresh xylene, and rehydrated through graded ethanols. Antigen retrieval was performed in citrate buffer (pH 6.0), followed by 1 h room‐temperature blocking with 5% BSA and overnight incubation at 4°C with primary antibodies (details in Table S1). Sections were subsequently treated with HRP‐conjugated rabbit secondary antibody (1 h, RT), developed with DAB, counterstained with hematoxylin (30 s), rinsed in distilled water (5 min), dehydrated, and imaged using an inverted fluorescence microscope.

Five high‐power fields from the tumor hotspot area were randomly selected for immunohistochemical interpretation, and ImageJ software was used to quantitatively evaluate the immunohistochemical results based on staining intensity (negative‐1 score, low positive‐2 score, positive‐3 score, high positive‐4 score) and the percentage of positive staining area (0‐100%). The staining score (ranging from 1 to 4) was calculated by multiplying the staining intensity and the percentage of positive staining area. The average score of the five fields is used to represent the final score. The median staining score (2.5 score) was set as the cut‐off value, cases with the final score ≥ 2.5 were classified as high‐expression, while those with a final score < 2.5 were classified as low‐expression.

### 4.6 Immunofluorescence (IF)

Formalin‐fixed paraffin‐embedded (FFPE) endometrial carcinoma tissue sections (4–5 µm) were subjected to deparaffinization, rehydration, antigen retrieval (Tris‐EDTA pH 9.0, 100°C, 20 min), permeabilization (0.25% Triton X‐100/PBS), and blocking (3% BSA/PBS). Sections were co‐incubated overnight at 4°C with primary antibodies (details in Table S1) diluted in blocking solution. After washing (PBST), sections were stained with fluorophore‐conjugated secondary antibodies and DAPI (1 µg/mL; 5 min). Slides were mounted using ProLong Diamond Antifade Mountant and imaged by confocal microscopy.

### 4.7 Western Blotting Analysis

RIPA lysis buffers with 1% phenylmethanesulfonylfluoride fluoride (PMSF) were used to extract the cellular proteins. Then, 10% SDS‐PAGE gels were prepared using the PAGE Gel Fast Preparation Kit (PG112, Epizyme Biomedical Technology, Shanghai, China). Equal amounts (20 µg) of protein were determined by the BeyoBCA Rapid Protein Assay Kit (P0398M, Beyotime Biotechnology, Shanghai, China) and separated using 10% SDS‐PAGE gels. The proteins were then transferred to PVDF membranes (0.45µm, Millipore, MA, USA). The membranes were blocked with 5% nonfat milk (BS102, Biosharp, Hefei, China) for 1 h at room temperature and then incubated with primary antibody (diluent) (details in Table S1) at 4°C for 12 h. Next, the membranes were incubated at room temperature for 1 h in anti‐rabbit (1:10000, SA00001‐2, Proteintech, Wuhan, China) or anti‐mouse (1:10000, SA00001‐1, Proteintech, Wuhan, China) secondary antibodies. Finally, the enhanced chemiluminescence (ECL) kit (12045‐D50, Advansta, USA) was utilized to visualize the protein bands.

### 4.8 Co‐Immunoprecipitation (CoIP)

After washing an adequate number of cells three times with ice‐cold PBS, cell lysis was performed using NP‐40 lysis buffer containing protease inhibitors, followed by 30 min incubation on ice. The lysate was centrifuged at 12 000 g for 15 min at 4°C, and the supernatant was divided into three portions: 10% for Input controls, 70% for target protein immunoprecipitation (IP), and 20% for IgG controls. The proteins of the IP group and IgG group were separately incubated overnight at 4°C with either sufficient amount of target‐specific antibodies (details in Table S1) or negative control IgG antibodies (30000‐0‐AP, Proteintech, Wuhan, China). Subsequently, 10 µL of protein A/G magnetic beads (HY‐K0202, MCE, NJ, USA) were added to each immunocomplex mixture and incubated for 1–2 h at room temperature with gentle agitation. The antibody‐protein complexes were captured using a magnetic rack (2 min immobilization), washed thoroughly, and boiled in 1× SDS loading buffer. Finally, target protein enrichment was analyzed by Western blot comparing Input, IgG control, and IP groups.

### 4.9 RNA Extraction, Reverse Transcription, and Quantitative PCR (RT‐qPCR)

Total RNA was extracted from cells using Trizol Reagent (Invitrogen, CA, USA). The Agilent 2100 Bioanalyzer (Agilent, Santa Clara, CA, USA) was used to determine the quality of total RNA. cDNA was obtained from total RNA using RT Master Mix for qPCR II (HY‐K0511A, MCE, NJ, USA). Next, according to the manufacturer's instructions, the SYBR Premix Ex Tag Kit (DRR420A, Takara, Tokyo, Japan) and real‐time PCR system (Bio Rad, CA, USA) were used to detect mRNA expression. GAPDH was used as an internal control. The relative quantification method (2^−ΔΔCt^ method) was applied to compare and analyze the quantitative results, and all experiments were repeated three times. The primers used for RT‐qPCR are listed in Table S2.

### 4.10 Flow Cytometry

Adherent cells or clustered ECSCs were digested into a single‐cell suspension using trypsin and washed three times with phosphate buffered saline (PBS). Flow cytometry antibodies were added to the cell suspension according to the manufacturer's instructions to stain the cells at room temperature for 20 min. The following flow cytometry antibodies were used in this study: FITC Mouse Anti‐Human CD44 (560977, Biolegend, CA, USA) and PE Mouse Anti‐Human CD133 (566593, Biolegend, CA, USA). After sufficient staining, the cells were washed three times with PBS, and then resuspended to 10^6^ cells/200µL. The cells were analyzed using the CytoFLEX LX flow cytometer (Beckman Coulter Life Sciences). FlowJo software was used for data analysis and graphical output.

### 4.11 Lentivirus Construction and Cell Transfection

Lentiviral vectors for constitutive knockdown of IGF2BP3/METTL3/HMGA2/AARS1, overexpression of IGF2BP3/HMGA2/AARS1, and expression of wild‐type and mutant IGF2BP3 (including IGF2BP3‐K266R, K272R, K280R, K440R, K450R, K475R, K508R, and K552R variants), as well as wild‐type/mutant HMGA2, were procured from Genechem (Shanghai) and LeapWal Biotech (Hunan). To overcome inherent lentiviral resistance in stem cells, parental EC cells were transduced at logarithmic growth phase by seeding 5 × 10^4^ cells/well in 6‐well plates; at 30% confluence, cells were incubated with viral particles complexed with HiTransG A enhancer (MOI = 20) for 16 h at 37°C. Following supernatant replacement with fresh RPMI‐1640/10% FBS, stable pools were selected using 2 µg/mL puromycin for 14 days. Lentiviral ECSCs were then induced by culturing in serum‐free DMEM/F12 supplemented with 20 ng/mL hEGF, 10 ng/mL hFGF, and B27. Transduction efficiency (>85% requirement) was monitored via GFP co‐expression throughout the process, with successful IGF2BP3/HMGA2 manipulation ultimately verified by RT‐qPCR and immunoblotting. The oligonucleotide sequences are shown in Table S3.

### 4.12 RNA m6A Quantification

According to the manufacturer's instructions, the EpiQuik m6A RNA Methylation Quantification Kit (P‐9005, Epigentek, NY, USA) was used to detect the m6A modification level of total RNA in the samples. Briefly, total RNA was extracted from cell or tissue samples using Trizol (Invitrogen, CA, USA), and the quality of RNA was assessed using the Agilent 2100 Bioanalyzer (Agilent, Santa Clara, CA, USA). Then, 200 ng RNA and m6A standard were coated onto the detection well, and capture antibody solution and detection antibody solution were added into the well. By measuring the absorbance at a wavelength of 450 nm (OD450), the m6A level was quantified using a colorimetric method, and then calculated based on the standard curve.

### 4.13 L‐Lactate Quantification

Fresh tissue samples or cells were homogenized in 450 µL ice‐cold PBS (0.1 m, pH 7.4) at 1:9 (w/v). The homogenate was centrifuged at 12 000 g for 10 min at 4°C to remove insoluble debris, and the clear supernatant was deproteinized by ultrafiltration (per kit requirement). Total lactate concentration was subsequently quantified kinetically at 570 nm using the L‐Lactic Acid Assay Kit (ab65330, Abcam), with sample absorbance measured against a lactate standard curve following reagent incubation at 37°C for 30 min.

### 4.14 RNA Immunoprecipitation (RIP)

According to the manufacturer's instructions, the Magna RIP kit (17‐700, Millipore, MA, USA) was used to perform RIP assays. In short, 2 × 10^7^ cells were added to a lysis buffer containing protease and RNase inhibitors and lysed on ice for 30 min. The cell lysate was then divided into two parts, one part was used as the Input group for whole cell extraction, and the other part was used for subsequent immunoprecipitation (IP) treatment (IP group). For the IP group, cell lysate was incubated overnight with 10 µg IGF2BP3 antibody (ab177477, Abcam, MA, USA) and IgG antibody (control) at 4°C. Protein A/G magnetic beads were washed five times continuously with 0.1% Tween 20, then mixed with cell lysate antibody complexes and rotated continuously at 4°C for 6 h. Next, the RNA protein complex was washed five times with eluent and treated with proteinase K at 55°C for 1 h. RNA that binds to the IGF2BP3 protein was extracted and the RT‐qPCR was performed to detect the expression of HMGA2. The results were standardized (Input%) and the expression results of the IP group were compared with the IgG group to demonstrate whether IGF2BP3 significantly binds to HMGA2 mRNA. The primers for RIP‐PCR are listed in Table S2.

### 4.15 m6A Methylated RNA Immunoprecipitation (MeRIP)

According to the manufacturer's instructions, the Magna MeRIP The m6A kit (17‐10499, Millipore, MA, USA) was used for MeRIP assays. In short, the total RNA from the sample was extracted and lysed into approximately 100nt using the lysis buffer at high temperature. Termination buffer was added to the RNA, and the precipitate was collected. Then the cell lysate was divided into two parts, one part was used as the Input group for whole cell extraction, and the other part was used for subsequent immunoprecipitation (IP) treatment (IP group). 50 µL of protein A/G magnetic beads and 10 µg of m6A antibody or 10 µg IgG antibody (control) were added to IP buffer (150mm NaCl, 0.1% NP‐40, 10mm Tris HCl, pH 7.4) and incubated at room temperature for 1 h. 6 µg fragment mRNA was added to the antibody magnetic bead mixture and incubated at 4°C for 4 h on a rotator. After thorough washing, the immunoprecipitated mixture was digested with high concentration proteinase K, and the bound RNA was extracted using the phenol chloroform method and ethanol. RT‐qPCR was performed on the RNA obtained from MeRIP to detect the m6A modification level of HMGA2 (IgG as negative control). The primers for MeRIP‐PCR are listed in Table S2.

### 4.16 Chromatin Immunoprecipitation (ChIP)

According to the manufacturer's instructions, the Pierce Agarose ChIP Kit (Thermo, USA) was used for ChIP assays. In short, cells were cross‐linked with 1% formaldehyde solution at room temperature for 10 min. Subsequently, 1 mL of glycine buffer was added to stop the crosslinking reaction. Next, micrococcal nuclease treatment was applied to digest the nucleoprotein complex and generate DNA fragments. The supernatant containing DNA fragments was divided into two parts, one as the Input group and the other as the IP group. In the IP group, 10 µg of HMGA2 antibody or 10 µg of IgG antibody (control) was added and rotated overnight at 4°C to obtain protein‐chromatin complexes. Then, A/G Plus agarose was added to the protein‐chromatin complex and incubated at 4°C for 1 h. Then, the immunoprecipitation complex was eluted, the DNA fragment was purified for subsequent RT‐qPCR analysis. Results were normalized (Input%). Primers were carefully designed and synthesized to target the predicted binding sites within the promoter region, ensuring specificity for subsequent analysis. The primers for ChIP‐PCR are listed in Table S2.

### 4.17 RNA Stability Assay

ECSCs maintained under sphere‐forming conditions were dissociated, seeded at 5 × 10^4^ cells/well in ultra‐low attachment 6‐well plates (Corning), cultured for 24 h to ∼50% confluence, and treated with 5 µg/mL actinomycin D (ActD; A9415, Sigma, USA) to block transcription. Cells were harvested at defined intervals (0/2/4/6 h, *n* = 3). Total RNA was extracted and quantitative analysis of HMGA2 mRNA expression was performed using RT‐qPCR. The expression of HMGA2 mRNA was calculated in each group of cells during the corresponding time period and was normalized using GAPDH. The degradation rate of HMGA2 mRNA was calculated based on relevant literature, and the differences in HMGA2 mRNA stability were compared among different cell groups using GraphPad Prism 8.0.2 (GraphPad Software, La Jolla, CA).

### 4.18 Protein Stability Assay

ECSCs from distinct treatment groups were treated with 40 µm cycloheximide. At 0, 24, and 48 h post‐treatment, total protein was extracted using RIPA lysis buffer supplemented with protease inhibitors. The lysates were centrifuged at 12 000g for 10 min at 4°C, and the supernatants were transferred to new microcentrifuge tubes. Protein concentrations were determined using the BCA assay with bovine serum albumin standards, followed by Western blot analysis to assess IGF2BP3 expression levels across groups. The protein half‐life of IGF2BP3 was calculated based on its time‐dependent degradation profile.

### 4.19 Luciferase Reporter Assay

The plasmid vectors of HMGA2 Wt/Mut, SOX2 Wt/Mut, OCT4 Wt/Mut, and CD44 Wt/Mut were purchased from Genecreate Biotechnology Co., Ltd (Wuhan, China). According to the manufacturer's instructions, the Dual‐Lumi Luciferase Reporter Gene Assay Kit (RG088S, Beyotime Biotechnology, Shanghai, China) was used for luciferase reporter assays. In short, 2 × 10^5^ cells were inoculated into a 24‐well plate and cultured for 24 h. Then the Wt/Mut plasmids were transfected into cells. After transfection for 12 h, the cells were re‐seeded into a 96‐well plate and incubated for 24 h. 100 µL of firefly luciferase detection reagent was added to the sample to react for 10 min, and the Multimode Microplate Reader was used to detect firefly luciferase (F‐luc). Then, 100 µL of renilla luciferase detection reagent was added to the sample to react for 10 min, and the renilla luciferase (R‐luc) was detected. R‐luc was used to achieve normalization of the F‐luc activity.

### 4.20 In Vitro Limiting Dilution Assay

ECSCs were seeded into ultra‐low adhesion 96‐well plates in a limiting dilution manner (100/50/20/10/5/1 per well) and suspension‐cultured for 7–10 days according to the ECSCs culture method described above. The formation of spheroids in each well was monitored, and the frequency of tumor‐initiating cells was calculated using extreme limiting dilution analysis (ELDA).

### 4.21 Sphere Formation Assay

ECSCs were induced following established protocols and cultured for 7 days in 6‐well low‐attachment plates under standard conditions (37°C, 5% CO_2_). Sphere formation was monitored daily via bright‐field microscopy (Olympus BX53F, Japan), with quantification performed by subjecting plates to horizontal shaking (100 rpm, 30 s) for uniform cellular distribution prior to imaging. Five randomly selected central fields of view per well were captured at 100× magnification using a calibrated microscopy system (Nikon Eclipse Ti; NIS‐Elements BR v4.60.00), followed by morphometric analysis quantifying: (1) numerical density derived from sphere counts per field area, and (2) sphere dimensions reporting mean maximum Feret's diameter (µm) measured through calibrated imaging software. All measurements were performed in triplicate wells across three independent biological replicates.

### 4.22 Poly (A) Tail‐Length Assay (PAT Assay)

The poly(A) tail dynamics of HMGA2 transcripts were quantified using the USB Poly(A) Tail‐Length Assay Kit (764551KT, Thermo, USA) per the manufacturer's protocol. Briefly, total RNA isolated from cell lysates via Trizol Reagent (Invitrogen) underwent DNase I treatment to eliminate genomic DNA, followed by enzymatic appending of a chimeric guanosine‐inosine anchor (G:I = 3:1) to existing 3'‐UTR poly(A) tails using recombinant poly(A) polymerase. First‐strand cDNA was then synthesized with SuperScript IV Reverse Transcriptase (18090010, Invitrogen) using the G/I anchor for priming, with parallel reactions containing gene‐specific HMGA2 3'‐UTR primers and universal reverse primers. Amplified products were resolved through 2% agarose electrophoresis for fragment length analysis.

### 4.23 In Vitro Lactylation Assay

Referring to previous similar studies [25], in vitro detection of AARS1‐mediated IGF2BP3 K280 lactylation was performed. The reaction buffer contained 50 mm Tris‐HCl (pH 7.6, ST776, Beyotime Biotech), 10 mm MgCl_2_ (ST269, Beyotime Biotech), 20 mm KCl (ST340, Beyotime Biotech), 1 mm DTT (ST041, Beyotime Biotech), 5%–10% glycerol (ST1348, Beyotime Biotech), 10 mm L‐lactate (L6402, Sigma), and 10 mm ATP (D7378, Beyotime Biotech). Purified recombinant AARS1 (0.5 mg/mL) was incubated with an equal concentration of wild‐type or K280R mutant IGF2BP3 protein (Sangon Biotech) at 37°C for 1 h. After incubation, samples were boiled in 5× SDS loading buffer for denaturation. IGF2BP3 K280 lactylation levels were determined by immunoblotting to evaluate the in vitro catalytic activity of AARS1.

### 4.24 Xenograft Animal Models

4‐week‐old female BALB/c nude mice were implanted subcutaneously in the right flank with 1 × 10^6^ stably transfected ECSCs suspended in 100 µL of Matrigel (356237, Corning)/PBS (1:1). Tumor growth was monitored twice weekly by dual‐axis caliper measurements, and the tumor volume was calculated as (minimum diameter)^2^ × maximum diameter × 0.5 [44]. When tumors reached 100–120 mm^3^ (typically 3 days post‐inoculation), mice were uniformly treated with gemcitabine (100 mg/kg, intraperitoneal injection, twice weekly for 3 weeks) [45]. At the experimental endpoint (day 28 post‐implantation), mice were euthanized by CO_2_ asphyxiation followed by cervical dislocation. Excised tumors were weighed and processed for downstream analyses.

### 4.25 In Vivo Limiting Dilution Assay

Serial dilutions of transfected ECSCs (1 × 10^5^, 1 × 10^4^, 1 × 10^3^, and 1 × 10^2^ in 50 µL PBS/Matrigel) were injected subcutaneously into the right thigh of recipient mice. Tumor volume was recorded every week. After 8 weeks, the nude mice were euthanized, and subcutaneous tumors were removed. The frequency of tumor‐initiating cells was calculated by ELDA. Excised tumors underwent IHC evaluation for IGF2BP3, HMGA2, CD44, and CD133 expression.

### 4.26 High‐Throughput Sequencing Analyses

RNA sequencing (RNA‐seq) and m6A RNA immunoprecipitation sequencing (MeRIP‐seq) was performed in triplicate for ECSCs derived from Ishikawa cells and their parental Ishikawa counterparts by LC‐Bio Technologies (Hangzhou, China). Separately, HMGA2 chromatin immunoprecipitation sequencing (ChIP‐seq) and IGF2BP3 RNA immunoprecipitation sequencing (RIP‐seq) datasets for parental Ishikawa cells were obtained from LC‐Bio Technologies and SeqHealth Technology Co., Ltd (Wuhan, China), respectively.

For RNA‐seq and MeRIP‐seq data, raw reads were quality‐controlled using FastQC (v0.12.1) and aligned to the GRCh38.p13 reference genome with STAR (v2.7.10b). Differential gene expression analysis identified significantly regulated genes (|log_2_FC| ≥1.0; adjusted *p* <0.05) using edgeR, while m6A‐enriched peaks were similarly called with identical thresholds. Peak distribution was annotated using HOMER (v4.11), and RNA‐seq results were cross‐validated against MeRIP‐seq m6A modification patterns. Functional enrichment analysis of deregulated genes was performed using Metascape (2023 update) with KEGG/GO databases.

For RIP‐seq and ChIP‐seq datasets, significant binding targets of IGF2BP3 and HMGA2 were identified through edgeR‐based enrichment analysis (IP vs. input; |log_2_FC| ≥1.0, *p* <0.05). Putative binding motifs were inferred using the MEME Suite (v5.5.0).

### 4.27 Lactylome Profiling

Lactylome profiling was performed by SeqHealth Technology Co., Ltd (Wuhan, China) to characterize the genome‐wide landscape of lysine lactylation in ECSCs. Specifically, the global lactylome dataset in this study was generated from wild‐type Ishikawa parental cells and their stably derived ECSCs. In brief, proteins were extracted from cells, digested overnight with trypsin, and the resulting peptides were desalted and vacuum dried. Lactylated peptides were enriched from the total peptide mixture using anti‐L‐lactyllysine antibody‐conjugated agarose beads (WM102; Micrometer Biotech Company, Hangzhou), followed by elution and vacuum concentration. The dried peptides were reconstituted, desalted using C18 ZipTip columns, and re‐dried before spiking with iRT standards for data‐independent acquisition (DIA) mass spectrometry analysis. Data processing was performed in Spectronaut with the following parameters: trypsin as protease (max 2 missed cleavages), fixed modification Carbamidomethyl (C), variable modifications Oxidation (M), Acetyl (Protein N‐terminus), and Lactyl (K), and protein identification filtered at a false discovery rate (FDR) <1%.

### 4.28 Molecular Docking

The structure of IGF2BP3 (PDB ID: O00425) was retrieved from the AlphaFold protein structure database, from which the three‐dimensional coordinates of the K280 residue were extracted using PyMOL's GetBox plugin. A focused library of SDF‐formatted antineoplastic agents (ATC class L) was downloaded from DrugBank, with compound geometries optimized via the MMFF94s force field in RDKit. For molecular docking experiments with both wild‐type IGF2BP3 and the K280R mutant model (generated using Swiss‐PDB Viewer), hydrogen atoms, Gasteiger charges, and nonpolar hydrogens were added through AutoDock Tools (v1.5.7). Semi‐flexible docking was performed using AutoDock Vina (v1.1.2) with 5 independent runs per compound, sampling binding modes against both the wild‐type and mutant binding site centered at K280 coordinates. All protein‐ligand complexes were visually analyzed with PyMOL.

### 4.29 Molecular Dynamics Simulation

Molecular dynamics simulations were performed in GROMACS 2024.3 starting from docked protein‐ligand structures. The system used AMBER14SB for protein, GAFF for ligand, and TIP3P water in a cubic box (≥1.2 nm solute‐edge distance), neutralized with Na^+^/Cl^−^ ions. Sequential preparation included: (1) 50 000‐step steepest descent energy minimization (emtol = 1000 kJ/mol·nm; Verlet cutoff; rCoulomb = rvdw = 1.0 nm); (2) Stepwise equilibration: (a) 300‐ps NVT (v‐rescale thermostat, 310.15 K; heavy‐atom restraints; LINCS/Particle Mesh Ewald electrostatics; rCoulomb = rvdw = 1.2 nm), (b) 300‐ps NPT (Parrinello–Rahman barostat, 1.0 bar; refcoord_scaling = com); (3) Unrestrained 100‐ns NPT production at 310.15 K, recording every 50 ps (LINCS/PME; rCoulomb = rvdw = 1.2 nm; neighborlist update per 20 steps). Subsequent analyses comprised backbone RMSD, Rg, SASA, intermolecular H‐bonds (≤3.5 Å/≥135°), per‐residue RMSF (50–100 ns trajectory), and Boltzmann‐weighted free‐energy landscapes (Rg/RMSD histograms, 310.15 K). MM‐PBSA calculations used 500 snapshots (50–100 ns) with per‐residue decomposition (PBSA: dielectric constants 1/80; 0.15 m salt; grid spacing 2.0 Å; nonpolar γ = 0.0072 kcal·mol^−^^1^·Å^−^^2^).

### 4.30 Integrated Predictive Model Incorporating IGF2BP3‐K280lac for EC Recurrence

We developed and validated a prognostic nomogram for EC recurrence incorporating the novel IGF2BP3‐K280lac biomarker assessed via immunohistochemistry. Briefly, leveraging a training cohort (*n* = 574) from our institution and an independent validation cohort (*n* = 231) from two tertiary hospitals, we first evaluated associations between IGF2BP3‐K280lac and clinicopathological features alongside its prognostic value in EC. Following confirmation of IGF2BP3‐K280lac as an independent predictor of recurrence‐free survival (RFS) by multivariable Cox regression (HR = 3.791, *p* <0.001), the post‐translational modification signature was integrated with seven RFS‐significant clinicopathological predictors—age, FIGO stage, pathological type, myometrial invasion, lymphovascular space invasion (LVSI), CA125 level, and p53 expression—to construct the prediction model. Model performance underwent rigorous internal and external validation through calibration curves and time‐dependent receiver operating characteristic (ROC) analysis.

### 4.31 Statistics

Data analysis was performed using SPSS 26.0 (IBM, Chicago, USA), GraphPad Prism 8.0.2 (GraphPad Software, La Jolla, CA), and R 4.2.2 (http://www.r‐project.org). Normally distributed continuous variables are represented by mean + SD, and the *t*‐test was used for comparison between two groups, 1‐way ANOVA was used for comparison among multiple groups. Non‐normally distributed data were represented as median (P25, P75), and the Wilcox test was used for comparison between two groups, while the Kruskal Wallis test was used for comparison among multiple groups. Categorical variables were expressed as frequency (%), and comparisons between groups were performed using the chi‐square test or Fisher's exact test. All experiments included ≥3 biological replicates. *p* <0.05 was considered statistically significant.

## Author Contributions

**Chao Tong** and **Yong Fu**: conceptualization, methodology, project administration, writing – review & editing. **Peng Jiang**: methodology, data curation, formal analysis, writing‐ original draft preparation, writing – review & editing. **Yunfeng Zheng**, **Chenfan Tian**, **Yuan Tu**, **Xiaoxia Chang**, **Jiaxin Yu**, **Hangkun Yu**, **Chunxia Gong**, **Jie Xiong** and **Philip N. Baker**: data curation, formal analysis. all authors critically reviewed the paper and approved the final version.

## Ethics Approval and Consent to Participate

All animal procedures were approved by the Animal Care Committee of Chongqing Medical University (IACUC‐CQMU‐2024‐0993). Endometrial carcinoma tissue specimens were obtained from patients who provided written informed consent specifically for sample use in research. The study protocol strictly adhered to the Declaration of Helsinki and received ethical clearance from three independent institutional review boards: The First Affiliated Hospital of Chongqing Medical University (No. 2024‐310‐01), Chongqing Medical University Affiliated Children's Hospital (No. 2023‐002), and Chongqing Liangjiang New Area People's Hospital (No. 2024–117).

## Conflicts of Interest

The authors declare no conflicts of interest.

## Supporting information




**Supporting File**: advs77757‐sup‐0001‐SuppMat.docx.

## Data Availability

The data that support the findings of this study are available on request from the corresponding author. The data are not publicly available due to privacy or ethical restrictions.
