# Supplementary material for: A Metabolic‐Epigenetic Axis of AARS1/IGF2BP3‐K280 Lactylation Sustains Endometrial Cancer Stemness and Recurrence
Source: Adv Sci (Weinh). 2026 Sep 23:e77757. Online ahead of print. doi: 10.1002/advs.77757 (PMC13598689; doi:10.1002/advs.77757)
Supplement: Supplementary file 1 [file ADVS-9999-e77757-s001.docx]

**A Metabolic-Epigenetic Axis of AARS1/IGF2BP3-K280 Lactylation Sustains Endometrial Cancer Stemness and Recurrence**

**Peng Jiang^1^,** **Yunfeng Zheng^1^,** **Chenfan Tian^1^, Yuan Tu^1^,** **Xiaoxia Chang^1^, Jiaxin Yu^1^, Hangkun Yu****^1^, Chunxia Gong^2^, Jie Xiong^3^, Philip N. Baker^4^ Yong Fu^1^*, Chao Tong^5^***

The file includes:

Supplementary Methods

Supplementary Figures (Supplementary Fig.1~ Supplementary Fig.23)

Supplementary Tables (Supplementary Tab.1~ Supplementary Tab.5)

**Supplementary Methods**

**Inclusion and exclusion criteria, treatment methods, postoperative follow-up and the recurrence definition of EC patients**

The inclusion criteria for patients were as follows: (1) The patient underwent standard comprehensive staging surgery, including at least abdominal total hysterectomy + bilateral salpingo-oophorectomy + lymph node assessment (lymph node assessment mainly includes pelvic lymph node resection ± paraaortic lymph node resection); (2) The final pathological diagnosis of the patient after surgery was endometrial cancer, and the pathological stage was FIGO stage I-III (Most stage IV patients did not receive surgical treatment and were unable to obtain postoperative pathological specimens. Meanwhile, stage IV patients had distant metastasis before treatment, which affected the subsequent assessment of patient recurrence. Therefore, stage IV patients were not included in this study). The exclusion criteria were as follows: (1) Patients with incomplete medical records; (2) Receiving adjuvant therapy before surgery; (3) Patients with other malignancies; (4) Loss of follow-up.

All patients were recommended to receive corresponding adjuvant treatment after surgery according to corresponding guidelines and multidisciplinary discussions. The implementation of the final adjuvant treatment plan was driven by the professional advice of clinical doctors and the personal wishes of patients, including follow-up, radiotherapy alone, chemotherapy alone, and the combination of radiotherapy and chemotherapy (chemoradiotherapy).

The follow-up plan of patients was carried out according to the corresponding guidelines: in short, follow-up was performed every 3 months for the ﬁrst 2 years after surgery, every 6 months for the next 3 years, and annually thereafter. The follow-up plans included regular physical examination and necessary auxiliary examinations, including serological examination (screening of tumor markers), imaging examination (B-ultrasound, CT, PET-CT, and MRI), and pathological biopsy. Recurrence-free survival (RFS) was deﬁned as the time from the surgical date to the conﬁrmed recurrence date, and overall survival (OS) was deﬁned as the time from the surgical date to the death. The follow-up deadline for this study was October 2024, and each patient has been guaranteed a follow-up period of more than 3 years.

Recurrence of EC was defined as local recurrence (including vaginal stump recurrence, central pelvic region recurrence) and distant metastases (including upper para-aortic lymph node metastases, peritoneal metastases, and metastases to other organs), which was confirmed by two or more gynecological oncologists based on the above examination and strive to obtain pathological support.

**Supplementary Figures**

**Supplementary Fig. 1 High tumor stemness was associated with EC recurrence.**

(A-D) TCGA/GTEx analysis: The distribution of stemness scores in EC (vs normal endometrium) (A), different stages (B), different pathological grades (C), and recurrent cases (vs non-recurrent cases) (D); (E-F) Reduced RFS (E) and OS (F) of EC patients with high stemness scores in UCEC cohort from the TCGA database; (G) IHC assay of CD44 and CD133 of EC patients from institutional cohorts; (H) The expression distribution of CD44 and CD133 in recurrent (n=76) and non-recurrent cases (n=498) from institutional cohorts based on IHC assay; (I-J) Distribution of RFS (I) and OS (J) of EC patients with different CD44 and CD133 expression states detected by IHC. Notes: (A, D) used Wilcox test; (B-C) used Kruskal Wallis test; (E-F) and (I-J) used Log-rank test; (H) used Chi-square test. Data was presented as mean±SD. *, P < 0.05; **, P < 0.01; ***, P < 0.001; ****, P < 0.0001.

**Supplementary Fig. 2 Culture and identification of ECSCs.**

(A) Spheroid formation (days 3, 5, 7) in ECSCs from Ishikawa, HEC1-A, RL952 lines and patient-derived ECCs; (B-E) Upregulation of stemness markers (CD44, CD133, SOX2, OCT4, NANOG) in Ishikawa/patient-derived ECSCs vs. parental cells by RT-qPCR (B-C) and WB (D-E); (F-G) Flow cytometry analysis of CD44/CD133 expression in ECSCs vs. parental cells. Notes: (B-C) used 1-way ANOVA analysis. All experiments were repeated three times. Data was presented as mean±SD. *, P < 0.05; **, P < 0.01; ***, P < 0.001; ****, P < 0.0001.

**Supplementary Fig. 3 Glycolytic activation correlates with elevated lactylation in recurrent and high-stemness endometrial cancer.**

(A–B) Glycolysis scores are significantly increased in recurrent tumors and high-stemness EC cases from the TCGA-UCEC cohort; (C–D) The expression of core glycolytic enzymes (GLUT1, HK2, and LDHA) is upregulated in recurrent EC tissues and ECSCs; (E) A positive correlation between glycolysis score and lactylation score is observed in EC samples. Notes: (A-B) used Wilcox test; (E) used Pearson correlation analysis. Data was presented as mean±SD. *, P < 0.05; **, P < 0.01; ***, P < 0.001; ****, P < 0.0001.

**Supplementary Fig. 4 Lactylation-mediated activation of m6A methylation**

(A) Distribution of m6A scores in high/low-lactylation score patients; (B) Correlation between lactylation and m6A modification scores; (C) Lactate treatment (20 μM) significantly increased total m6A levels in ECSCs; (D) The mRNA level of IGF2BP3 in ECSCs with/without lactate treatment (qRT-PCR). Notes: (A) used Wilcox test; (B) used Pearson correlation analysis; (C-D) used 1-way ANOVA analysis. (C-D) were repeated three times. Data was presented as mean±SD. *, P < 0.05; **, P < 0.01; ***, P < 0.001; ****, P < 0.0001.

**Supplementary Fig. 5 Proteomic lactylome analysis of ECSCs^Ishikawa^ reveals multi-site lactylation of IGF2BP3**

**Supplementary Fig. 6 MeRIP-seq displayed the distribution of m6A modifications in ECSCs and parental EC cells.**

(A) The bar chart showed the number of m6A modifications significantly downregulated and upregulated in ECSCs ^Ishikawa^ compared to the parental Ishikawa cells; (B-C) MeRIP-seq suggested that m6A modifications were mainly distributed at the 3 'UTR end of mRNA in both ECSCs ^Ishikawa^ and parental Ishikawa cells.

**Supplementary Fig. 7 IGF2BP3-target gene correlations (JMJD1C, CDK14, ETV6, PBX3, BIRC3) in TCGA-UCEC cohort.**

**Supplementary Fig. 8 (A-B) Validation of IGF2BP3 knockdown efficiency by qRT-PCR/WB and (C-D) regulatory effect of IGF2BP3 overexpression on HMGA2 by qRT-PCR/WB**

Notes: (A-C) used 1-way ANOVA analysis. All experiments were repeated three times. Data was presented as mean±SD. *, P < 0.05; **, P < 0.01; ***, P < 0.001; ****, P < 0.0001.

**Supplementary Fig. 9 HMGA2 overexpression links to adverse pathology and recurrence in endometrial cancer (EC).**

(A-D) HMGA2 expression in TCGA/GTEx: EC vs. normal endometrium (A), across stages (B), grades (C), and molecular subtypes (D); (E) IHC validation: HMGA2 in normal vs. EC tissues (HPA database); (F-G) Survival outcomes: RFS (F) and OS (G) by HMGA2 expression (TCGA-UCEC); (H-J) IHC cohorts (n=574): Recurrence (H), RFS (I), and OS (J) curves by HMGA2 status. Notes: (A) used Wilcox test; (B-D) used Kruskal Wallis test; (F-J) used Log-rank test. Data was presented as mean±SD. *, P < 0.05; **, P < 0.01; ***, P < 0.001; ****, P < 0.0001.

**Supplementary Fig. 10 HMGA2 positively regulated EC tumor stemness in vitro.**

(A) Distribution of stemness scores in patients with high or low expression of HMGA2 (UCEC cohort from TCGA database); (B) Correlation analysis between HMGA2 expression and stemness scores (UCEC cohort from TCGA database); (C-D) RT-qPCR (C) and WB (D) were used to detect the expression of HMGA2 in ECSCs and parental EC cells; (E) Validation of the correlation between HMGA2 and tumor stemness markers (CD44 and CD133) based on IHC assay (institutional cohorts, n=574); (F-G) RT-qPCR (F) and WB (G) were used to validate the knockdown efficiency of HMGA2; (H-I) Assessment of stemness markers (CD44, CD133, SOX2, OCT4, and NANOG) expression after HMGA2 knockdown in different EC cell lines at protein (H) and mRNA (I) levels; (J) Sphere formation assay in HMGA2 deficient EC cells; (K-N) In vitro limiting dilution assay of HMGA2 deficient EC cells. Notes: (A) used Wilcox test; (C) used unpaired t test; (F, I-J) used 1-way ANOVA analysis. (B, E) used Pearson correlation analysis. (C-D, F-N) were all repeated three times. Data was presented as mean±SD. *, P < 0.05; **, P < 0.01; ***, P < 0.001; ****, P < 0.0001. sh#1, shHMGA2#1; sh#2, shHMGA2#2.

**Supplementary Fig. 11 Tumor sphere-initiating frequency analysis of in vitro limiting dilution assays for ECSCs under different treatments.**

**Supplementary Fig. 12 HMGA2 directly activates stemness gene transcription in EC**

(A) HMGA2 ChIP-seq peaks at target gene TSS (Ishikawa cells); (B) HMGA2-bound TSS regions with enriched A/T motifs; (C-E) HMGA2 ChIP-seq binding peaks in SOX2/OCT4/CD44 promoters; (F-H) Enhanced HMGA2 binding to stemness genes (SOX2/OCT4/CD44) in ECSCs vs. parental cells (ChIP-PCR); (I-K) Reduced HMGA2 binding to stemness genes upon HMGA2 knockdown (ChIP-PCR); (L-N) Schematics: Mutations in potential binding sites of SOX2/OCT4/CD44; (O-Q) A/T motif-dependent transcriptional activation verified by luciferase assay (WT vs. HMGA2-KD EC cells). Notes: (F-K, O-Q) used 1-way ANOVA analysis. All experiments were repeated three times. Data represent mean±SD. *, P < 0.05; **, P < 0.01; ***, P < 0.001; ****, P < 0.0001. TSS: transcriptional start site; SOX2/OCT4/CD44-Wt: Wildtype; Mut: Binding site mutation.

**Supplementary Fig. 13 CoIP-MS analysis of AARS1 reveals prominent enrichment of IGF2BP3 peptides (A) and characteristic MS/MS spectra of IGF2BP3 peptides (B-C)**

**Supplementary Fig. 14 Lactate treatment (20mM) enhances the protein–protein interaction between AARS1 and IGF2BP3 in ECSCs.**

**Supplementary Fig. 15 Association of AARS1 with stemness and recurrence in EC**
(A) Stemness score distribution in patients with high/low AARS1 expression (TCGA-UCEC); (B) Correlation between AARS1 expression and stemness scores (TCGA-UCEC); (C) AARS1 protein levels in ECSCs versus parental cells by WB; (D) WB analysis of AARS1 in non-recurrent versus recurrent EC patients; (E) Representative IHC staining of AARS1 in non-recurrent and recurrent cases; (F) Distribution of AARS1 IHC scores in non-recurrent (n=498) and recurrent (n=76) patient cohorts; (G-H) Recurrence-free survival (RFS, G) and overall survival (OS, H) curves stratified by AARS1 expression. Notes: (A) used Wilcox test; (B) used Pearson correlation analysis; (F) used unpaired t test; (G-H) used Log-rank test. (C-D) were repeated three times. Data was presented as mean±SD. *, P < 0.05; **, P < 0.01; ***, P < 0.001; ****, P < 0.0001.

**Supplementary Fig. 16 Quantitative analysis of relative IGF2BP3 protein stability in ECSCs ^Ishikawa^ with different treatments in Figure 7J.** Notes: 2-way ANOVA analysis were used and were repeated three times. Data was presented as mean±SD. *, P < 0.05; **, P < 0.01; ***, P < 0.001; ****, P < 0.0001.

**Supplementary Fig. 17 *In vitro* limiting dilution assays for ECSCs under different treatments.**

(A) Representative ELDA plots depicting extreme limiting dilution analysis, and (B) tumor sphere-initiating frequency analysis.

**Supplementary Fig. 18 Prognostic value of IGF2BP3-K280 lactylation in endometrial cancer**

(A) RFS and OS in patients stratified by IGF2BP3^K280-lac^ levels (IHC; institutional cohort, n=574); (B) ROC curves for recurrence/death prediction (institutional cohort, n=574); (C) Multivariable Cox regression of RFS/OS for EC (institutional cohort, n=574).

**Supplementary Fig. 19 Participant Flow Diagram for EC recurrence prediction model**

**Supplementary Fig. 20 Development and validation of endometrial cancer recurrence prediction model**

(A) Nomogram for predicting EC recurrence; (B) Calibration curves for internal (n=574) and external (n=231) validation; (C) Time-dependent ROC curves for internal (n=574) and external (n=231) validation; (D) Combined ROC analysis: Clinicopathological characteristics alone vs. IGF2BP3^K280-lac^ expression alone vs. integrated model.

**Supplementary Fig. 21 Molecular dynamics analysis of gemcitabine-IGF2BP3 complex**
(A) RMSF of residues; (B) Radius of gyration (Rg); (C) Hydrogen bonds at binding interface; (D) Solvent-accessible surface area (SASA); (E) Binding free energy evolution over time; (F) Enthalpic contributions to binding energy; (G) Simulation snapshot at 100 ns.

**Supplementary Fig. 22 Determination of the IC_50_ values of gemcitabine (treatment for 48h) in Ishikawa-derived ECSCs and patient-derived ECSCs.**

**Supplementary Fig. 23 Tumor volume (A) and weight (B) in subcutaneous xenografts across different treatment groups**

**Supplementary Tables**

**Supplementary Tab. 1 Information on primary antibodies used for IHC, WB, and IP experiments**

| **Antibody (IHC)** | **Dilution** | **Catalog #** | **Vendor** |
| --- | --- | --- | --- |
| AARS1 | 1:200 | 17394-1-AP | Proteintech |
| IGF2BP3 | 1:200 | 14642-1-AP | Proteintech |
| HMGA2 | 1:500 | 20795-1-AP | Proteintech |
| Pan-Klac | 1:200 | PTM-1401 | PTM Biolabs |
| CD44 | 1:500 | 15675-1-AP | Proteintech |
| CD133 | 1:4000 | 18470-1-AP | Proteintech |
| IGF2BP3^K280-lac^* | 1:200 | N/A | Youke Biotechnology |
| **Antibody (IF)** | **Dilution** | **Catalog #** | **Vendor** |
| IGF2BP3 | 1:200 | 14642-1-AP | Proteintech |
| IGF2BP3^K280-lac^* | 1:200 | N/A | Youke Biotechnology |
| **Antibody (WB)** | **Dilution** | **Catalog #** | **Vendor** |
| AARS1 | 1:1000 | 17394-1-AP | Proteintech |
| IGF2BP3 | 1:5000 | ab177477 | Abcam |
| HMGA2 | 1:2000 | ab97276 | Abcam |
| Pan-Klac | 1:1000 | PTM-1401 | PTM Biolabs |
| CD44 | 1:20,000 | 15675–1-AP | Proteintech |
| CD133 | 1:2000 | 18470–1-AP | Proteintech |
| SOX2 | 1:1000 | ab97959 | Abcam |
| OCT4 | 1:1000 | 11263-1-AP | Proteintech |
| NANOG | 1:1000 | 14295-1-AP | Proteintech |
| GAPDH | 1:50,000 | 60004-1-Ig | Proteintech |
| β-actin | 1:20,000 | 66009-1-Ig | Proteintech |
| HK2 | 1:10,000 | 22029-1-AP | Proteintech |
| GLUT1 | 1:4000 | 21829-1-AP | Proteintech |
| LDHA | 1:5000 | 19987-1-AP | Proteintech |
| IGF2BP3^K280-lac^* | 1:2000 | N/A | Youke Biotechnology |
| **Antibody (IP)** | **Dilution** | **Catalog #** | **Vendor** |
| AARS1 | N/A | 17394-1-AP | Proteintech |
| IGF2BP3 | N/A | ab177477 | Abcam |
| Pan-Klac | N/A | PTM-1401 | PTM Biolabs |
| Flag | N/A | 14793S | Cell Signaling Technology |

*Site-specific custom antibody against K280-lactylated epitope of IGF2BP3

**Supplementary Tab. 2 The Primers of RT-qPCR, RIP-PCR, MeRIP-PCR and ChIP-PCR**

| **Gene** | **Primer sequence for RT-qPCR** |
| --- | --- |
| IGF2BP3 | (Forward Primer) 5' ACACCTGATGAGAATGACCAAGT 3'  (Reverse Primer) 5' CTTCTGTTGTTGGTGCTGCTTTA 3' |
| HMGA2 | (Forward Primer) 5' CGGTGAGCCCTCTCCTAAG 3'  (Reverse Primer) 5' CTCCAGTGGCTTCTGCTTTC 3' |
| SOX2 | (Forward Primer) 5' TGGGTTCGGTGGTCAAGTC 3'  (Reverse Primer) 5' GCTCTGGTAGTGCTGGGACA 3' |
| OCT4 | (Forward Primer) 5' CAGTATCGAGAACCGAGTGAGA 3'  (Reverse Primer) 5' GATGTGGCTGATCTGCTGC 3' |
| NANOG | (Forward Primer) 5' ACCTATGCCTGTGATTTGTGG 3'  (Reverse Primer) 5' GGTTGTTTGCCTTTGGGAC 3' |
| CD44 | (Forward Primer) 5' GCAACCCTACTGATGATGACG 3'  (Reverse Primer) 5' TCTGTCTGTGCTGTCGGTGA 3' |
| CD133 | (Forward Primer) 5' TCAAGATACTTCAACGCACAGG 3'  (Reverse Primer) 5' GCACGATGCCACTTTCTCAC 3' |
| β-actin | (Forward Primer) 5' AGAAAATCTGGCACCACACCT 3'  (Reverse Primer) 5' GATAGCACAGCCTGGATAGCA 3' |
|  | **Primer sequence for RIP-PCR, MeRIP-PCR** |
| HMGA2 | (Forward Primer) 5' CAGGAGGAAACTGAAGAGACATC 3'  (Reverse Primer) 5' CAACTGCTGCTGAGGTAGAAATC 3' |
|  | **Primer sequence for ChIP-PCR** |
| SOX2 | (Forward Primer) 5' AGGTTAGTAAGGAACAAAACAATGC 3'  (Reverse Primer) 5' TATAAAGGGCTTAAACCATCAGG 3' |
| OCT4 | (Forward Primer) 5' CAGGGCTCTTTGTCCACTTTG 3'  (Reverse Primer) 5' AATTGATTATCCCTCATCTTTACTG 3' |
| CD44 | (Forward Primer) 5' TTGAGGTAAACTTGGTACATCTTGC 3'  (Reverse Primer) 5' GTCCCTTTCCAGCTAATTTAACATC 3' |

**Supplementary Tab. 3 The shRNA sequences of IGF2BP3 and HMGA2**

| **Gene** | **Target sequences** |
| --- | --- |
| shAARS1#1 | 5' GCTCCTCTATGACACCTATGG 3' |
| shAARS2#2 | 5' GGAAACTGGCCCAGCTGAAAT 3' |
| shAARS3#3 | 5' GCAAGGAGGCCAGATCTATGA 3' |
| shIGF2BP3#1 | 5' CGGTGAATGAACTTCAGAATT 3' |
| shIGF2BP3#2 | 5' GCAGTTGTAAATGTAACCTAT 3' |
| shIGF2BP3#3 | 5' GCACATTTAATTCCTGGATTA 3' |
| shHMGA2#1 | 5' AGTCCCTCTAAAGCAGCTCAA 3' |
| shHMGA2#2 | 5' CTCCTAAGAGACCCAGGGGAA 3' |
| shHMGA2#3 | 5' AACGGCCAAGAGGCAGACCTA 3' |
| shMETTL3 | 5' GGGCCCAAGTGCAAGAATTCT 3' |
| shNC | 5' TTCTCCGAACGTGTCACGT 3' |

**Supplementary Tab. 4 Distribution of different clinicopathological characteristics of EC patients in high and low expression group of IGF2BP3**

| **Variable** | **Total (n=574)** | **IGF2BP3^K280-lac^ expression** | | **p-value** |
| --- | --- | --- | --- | --- |
|  |  | Low (n=325) | High (n=249) |  |
| **Age (yrs)**  [Mean (±SD)] | 53.87 (±9.46) | 53.06 (±9.80) | 54.92 (±8.92) | **0.020** |
| **BMI** (kg/m2)  [Mean (±SD)] | 24.53 (±3.78) | 24.40 (±3.66) | 24.69 (±3.92) | 0.367 |
| **FIGO stage** |  |  |  |  |
| I | 390 (67.9%) | 233 (71.7%) | 157 (63.1%) | 0.088 |
| II | 61 (10.6%) | 31 (9.5%) | 30 (12.0%) |  |
| III | 123 (21.4%) | 61 (18.8%) | 62 (24.9%) |  |
| **Pathological type** |  |  |  |  |
| G1 and G2 EEC | 421 (73.3%) | 257 (79.1%) | 164 (65.9%) | **＜0.001** |
| G3 EEC | 61 (10.6%) | 31 (9.5%) | 30 (12.0%) |  |
| NEEC | 92 (16.0%) | 37 (11.4%) | 55 (22.1%) |  |
| **Myometrial invasion** |  |  |  |  |
| ＜1/2 | 415 (72.3%) | 251 (77.2%) | 164 (65.9%) | **0.003** |
| ≥1/2 | 159 (27.7%) | 74 (22.8%) | 85 (34.1%) |  |
| **Cervical stromal invasion** |  |  |  |  |
| No | 492 (85.7%) | 280 (86.2%) | 212 (85.1%) | 0.731 |
| Yes | 82 (14.3%) | 45 (13.8%) | 37 (14.9%) |  |
| **LVSI** |  |  |  |  |
| Negative | 429 (74.7%) | 260 (80.0%) | 169 (67.9%) | **＜0.001** |
| Positive | 145 (25.3%) | 65 (20.0%) | 80 (32.1%) |  |
| **P53 expression** |  |  |  |  |
| Normal | 372 (64.8%) | 215 (66.2%) | 157 (63.1%) | 0.441 |
| Abnormal | 202 (35.2%) | 110 (33.8%) | 92 (36.9%) |  |
| **Adjuvant treatment** |  |  |  |  |
| Follow-up | 204 (35.5%) | 117 (36.0%) | 87 (34.9%) | 0.922 |
| Radiotherapy | 184 (32.1%) | 102 (31.4%) | 82 (32.9%) |  |
| Chemotherapy | 19 (3.3%) | 12 (3.7%) | 7 (2.8%) |  |
| Chemoradiotherapy | 167 (29.1%) | 94 (28.9%) | 73 (29.3%) |  |
| **Recurrence** |  |  |  |  |
| No | 490 (85.4%) | 297 (91.4%) | 193 (77.5%) | **＜0.001** |
| Yes | 84 (14.6%) | 28 (8.6%) | 56 (22.5%) |  |
| **Death** |  |  |  |  |
| No | 518 (90.2%) | 311 (95.7%) | 207 (83.1%) | **0.002** |
| Yes | 56 (9.8%) | 14 (4.3%) | 42 (16.9%) |  |
| **RFS time** (months)  [Median (P25, P75)] | 46.00 (38.00,60.25) | 46.00(39.00,58.00) | 45.00(35.00,62.00) | 0.205 |
| **OS time** (months)  [Median (P25, P75)] | 47.00 (38.00,62.00) | 46.00(39.00,59.50) | 47.00(37.00,62.50) | 0.858 |
| **Abbreviations:** BMI, body mass index; FIGO, International Federation of Gynecology and Obstetrics; EEC, endometrioid carcinoma; LVSI, lymph vascular space invasion; | | | | |

**Supplementary Tab. 5 Distribution of baseline data for training and validation cohorts**

| **Variable** | **Training cohort (n=574)** | **Validation cohort (n=231)** | **p-value** |
| --- | --- | --- | --- |
| **Age (yrs)**  [Mean (±SD)] | 53.87 (±9.46) | 53.21 (±8.94) | 0.364 |
| **BMI** (kg/m2)  [Mean (±SD)] | 24.53 (±3.78) | 24.90 (±4.12) | 0.217 |
| **FIGO Stage** |  |  |  |
| I | 390 (67.9%) | 166 (71.9%) | 0.551 |
| II | 61 (10.6%) | 22 (9.5%) |  |
| III | 123 (21.4%) | 43 (18.6%) |  |
| **Pathological type** |  |  |  |
| G1 and G2 EEC | 421 (73.3%) | 179 (77.5%) | 0.354 |
| G3 EEC | 61 (10.6%) | 24 (10.4%) |  |
| NEEC | 92 (16.0%) | 28 (12.1%) |  |
| **Myometrial invasion** |  |  |  |
| ＜1/2 | 415 (72.3%) | 163 (70.6%) | 0.620 |
| ≥1/2 | 159 (27.7%) | 68 (29.4%) |  |
| **Cervical stromal invasion** |  |  |  |
| No | 492 (85.7%) | 205 (88.7%) | 0.254 |
| Yes | 82 (14.3%) | 26 (11.3%) |  |
| **LVSI** |  |  |  |
| Negative | 429 (74.7%) | 180 (77.9%) | 0.341 |
| Positive | 145 (25.3%) | 51 (22.1%) |  |
| **P53 expression** |  |  |  |
| Normal | 372 (64.8%) | 160 (69.3%) | 0.227 |
| Abnormal | 202 (35.2%) | 71 (30.7%) |  |
| **IGF2BP3^K280-lac^ expression** |  |  |  |
| Low | 325 (56.6%) | 144 (62.3%) | 0.137 |
| High | 249 (43.4%) | 87 (37.7%) |  |
| **Adjuvant treatment** |  |  |  |
| Follow-up | 204 (35.5%) | 93 (40.3%) | 0.314 |
| Radiotherapy | 184 (32.1%) | 59 (25.5%) |  |
| Chemotherapy | 19 (3.3%) | 9 (3.9%) |  |
| Chemoradiotherapy | 167 (29.1%) | 70 (30.3%) |  |
| **Recurrence** |  |  |  |
| No | 490 (85.4%) | 191 (82.7%) | 0.340 |
| Yes | 84 (14.6%) | 40 (17.3%) |  |
| **Death** |  |  |  |
| No | 518 (90.2%) | 199 (86.1%) | 0.092 |
| Yes | 56 (9.8%) | 32 (13.9%) |  |
| **RFS time** (months)  [Median (P25, P75)] | 46.00 (38.00,60.25) | 48.00 (37.00,62.00) | 0.543 |
| **OS time** (months)  [Median (P25, P75)] | 47.00 (38.00,62.00) | 49.00 (38.00,63.00) | 0.425 |
